# The Endothelial Transcription Factor ERG Promotes Vascular Stability and Growth through Wnt/β-Catenin Signaling

**DOI:** 10.1016/j.devcel.2014.11.016

**Published:** 2015-01-12

**Authors:** Graeme M. Birdsey, Aarti V. Shah, Neil Dufton, Louise E. Reynolds, Lourdes Osuna Almagro, Youwen Yang, Irene M. Aspalter, Samia T. Khan, Justin C. Mason, Elisabetta Dejana, Berthold Göttgens, Kairbaan Hodivala-Dilke, Holger Gerhardt, Ralf H. Adams, Anna M. Randi

**Affiliations:** 1 National Heart and Lung Institute (NHLI) Vascular Sciences, Hammersmith Hospital, Imperial College London, London W12 0NN, UK; 2 Centre for Tumour Biology, Barts Cancer Institute – a CR-UK Centre of Excellence, John Vane Science Centre, Queen Mary University of London, Charterhouse Square, London EC1M 6BQ, UK; 3 Vascular Biology Laboratory, London Research Institute, Cancer Research UK, London WC2A 3PX, UK; 4 FIRC Institute of Molecular Oncology Foundation, IFOM, 20139 Milan, Italy; 5 Department of Haematology, Wellcome Trust and MRC Cambridge Stem Cell Institute and Cambridge Institute for Medical Research, University of Cambridge, Cambridge CB2 0XY, UK; 6 Department of Tissue Morphogenesis, Max Planck Institute for Molecular Biomedicine and Faculty of Medicine, University of Münster, D-48149 Münster, Germany

## Abstract

Blood vessel stability is essential for embryonic development; in the adult, many diseases are associated with loss of vascular integrity. The ETS transcription factor ERG drives expression of VE-cadherin and controls junctional integrity. We show that constitutive endothelial deletion of ERG (*Erg*^*cEC-KO*^) in mice causes embryonic lethality with vascular defects. Inducible endothelial deletion of ERG (*Erg*^*iEC-KO*^) results in defective physiological and pathological angiogenesis in the postnatal retina and tumors, with decreased vascular stability. ERG controls the Wnt/β-catenin pathway by promoting β-catenin stability, through signals mediated by VE-cadherin and the Wnt receptor Frizzled-4. Wnt signaling is decreased in ERG-deficient endothelial cells; activation of Wnt signaling with lithium chloride, which stabilizes β-catenin levels, corrects vascular defects in *Erg*^*cEC-KO*^ embryos. Finally, overexpression of ERG in vivo reduces permeability and increases stability of VEGF-induced blood vessels. These data demonstrate that ERG is an essential regulator of angiogenesis and vascular stability through Wnt signaling.

## Highlights

• Inducible deletion of endothelial ERG in mice causes defective angiogenesis
• ERG controls vascular stability through Wnt/β-catenin signaling
• β-catenin activation rescues the angiogenic defect in vivo in ERG-deficient mice
• Overexpression of ERG in vivo stabilizes VEGF-induced angiogenesis

## Graphical Abstract

**Figure:**
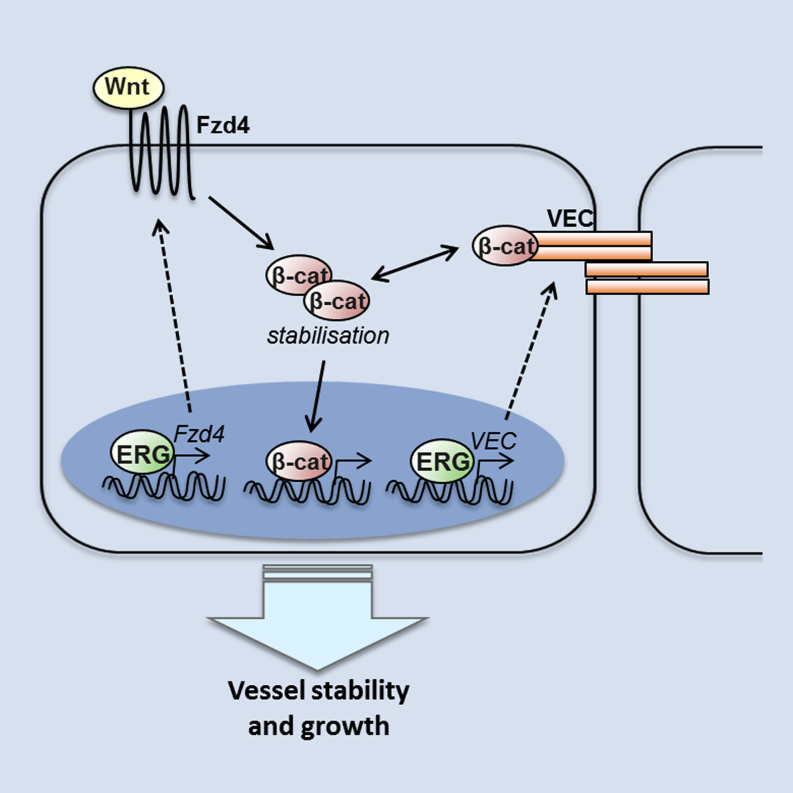

## Teaser

Birdsey, Shah et al. show that the endothelial ETS factor ERG controls Wnt/β-catenin signaling by promoting β-catenin stability, through pathways mediated by VE-cadherin and the Wnt receptor Frizzled-4. In vivo, ERG overexpression stabilizes VEGF-dependent angiogenesis. Thus, ERG is an essential regulator of angiogenesis and vascular stability through Wnt signaling.

## Introduction

Angiogenesis is essential during embryogenesis and is a critical component of many diseases. Coordination of growth and stability signals is required for effective angiogenesis (Jain, 2003). Diseases such as cancer, diabetic retinopathy, and vascular malformations are associated with vascular instability, which causes increased permeability and edema, excessive and/or dysfunctional angiogenesis, and hemorrhage. New strategies that target the maturation of blood vessels and restore vascular integrity could therefore have important therapeutic implications.

Multiple interactions at endothelial cell-cell junctions control vascular integrity. Crucial among these are the adhesion molecule vascular endothelial (VE)-cadherin and its intracellular partner β-catenin, an essential component of the canonical Wnt pathway (reviewed in Dejana, 2010). β-catenin is a multifunctional protein that can act as a scaffold between VE- and N-cadherins and the actin cytoskeleton, and as a coregulator for the T cell factor (TCF)/lymphoid enhancer-binding factor transcription factor complex. β-catenin levels are controlled by phosphorylation through a cytoplasmic degradation complex (reviewed in Reis and Liebner, 2013; Dejana, 2010). In the presence of Wnt ligands, which bind to a receptor complex containing members of the Frizzled (Fzd) family, the degradation complex is inactivated; β-catenin is stabilized and translocates to the nucleus to promote transcription. In the vasculature, the Wnt/β-catenin pathway controls vascular stability through remodeling, junction assembly, and pericyte recruitment (reviewed in Reis and Liebner, 2013; Dejana, 2010; Franco et al., 2009).

The ETS transcription factor family is implicated in vascular development and angiogenesis (reviewed in Randi et al., 2009). The ETS related gene (ERG), expressed throughout the life of the endothelium, regulates multiple pathways involved in vascular homeostasis and angiogenesis, such as monolayer integrity, endothelial permeability, and survival (Birdsey et al., 2008, 2012; Yuan et al., 2012). Previous studies have indicated a role for ERG in vascular development and angiogenesis (Baltzinger et al., 1999; Birdsey et al., 2008; Liu and Patient, 2008). Vijayaraj et al. reported that global deletion of a subset of endothelial ERG isoforms in mice results in defects in vascular and cardiac morphogenesis, causing embryonic lethality (Vijayaraj et al., 2012). The mechanisms through which ERG controls blood vessel formation are still unclear, and its therapeutic potential is unexplored.

In this study, we use genetic lineage-specific mouse models and multiple in vitro models to show that ERG promotes vascular growth and stability, through control of the canonical Wnt/β-catenin pathway. Crucially, we demonstrate that overexpression of ERG in vivo enhances vascular endothelial growth factor (VEGF)-dependent angiogenesis and promotes stability of VEGF-induced new blood vessels.

## Results

### Endothelial ERG Is Required for Vascular Development, Angiogenesis, and Tumor Growth

**Figure 1:**
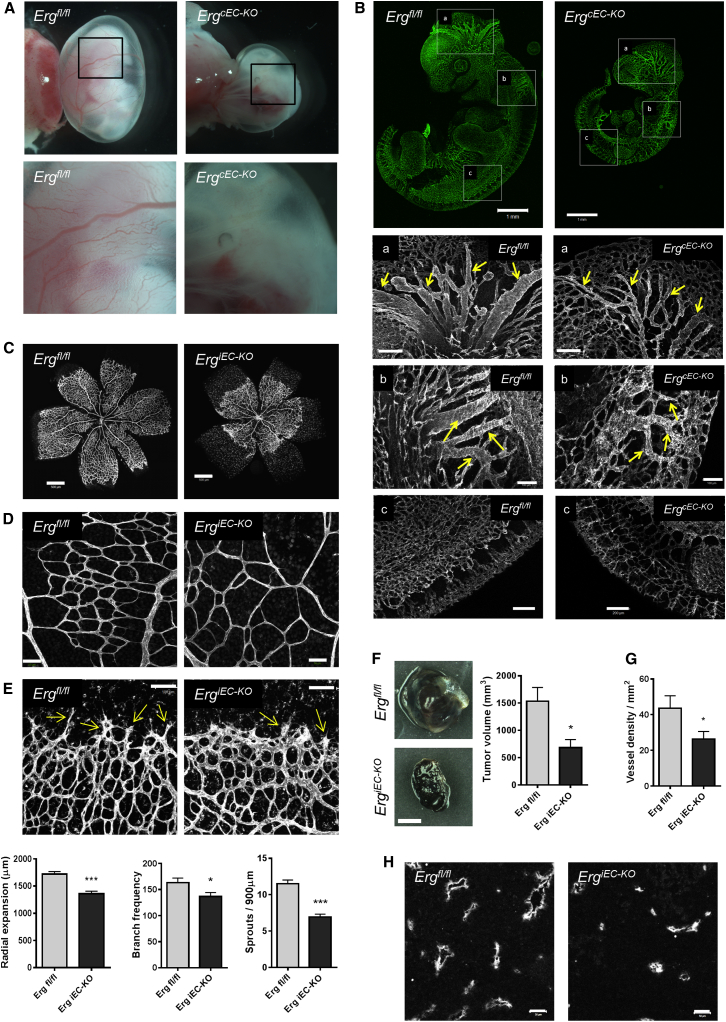
ERG Is Required for Vascular Development, Physiological Postnatal Angiogenesis, and Pathological Tumor Angiogenesis (A) Representative whole mount images of E10.5 *Erg*^*fl/fl*^ and *Erg*^*cEC-KO*^ embryo yolk sacs (magnification × 0.7). Bottom panel shows higher magnification of yolk sacs (magnification × 2). (B) Endomucin staining of blood vessels in E10.5 *Erg*^*fl/fl*^ and *Erg*^*cEC-KO*^ embryos; scale bars, 1 mm. Higher magnification of embryos shows vessel detail in the head region (panels a and b; scale bars, a, 200 μm and scale bars, b, 100 μm) and the trunk (panel c, scale bar, 200 μm). (C) Isolectin B4 staining of postnatal day 6 retinas from *Erg*^*fl/fl*^ and *Erg*^*iEC-KO*^ mice, showing vascular progression, scale bar, 500 μm; quantification (n = 6). (D) Vascular density of isolectin B4 stained branches in the central plexus, scale bar, 50 μm; quantification (n = 6). (E) EC sprouts at the angiogenic front (arrows), scale bar, 100 μm; quantification (n = 6). (F) Representative images of B16F0 tumors which were grown for 14 days on adult *Erg*^*iEC-KO*^ and *Erg*^*fl/fl*^ mice, scale bar, 2 mm; tumor volume was quantified (n = 6). (G and H) Panels show endomucin staining of blood vessels in B16F0 tumors and the quantification of the number of endomucin-positive vessels, (n = 6), scale bar, 50 μm. All graphical data are ± SEM, ^∗^p < 0.05, and ^∗∗∗^p < 0.001. See also Figure S1.

To investigate the role of endothelial ERG in vivo, we used a *Cre/loxP* strategy. The floxed allele of *Erg* was obtained by inserting two loxP sites flanking exon 6 (Figure S1A available online). Constitutive endothelial-specific deletion of *Erg* was achieved by breeding floxed *Erg* mice with mice expressing the *Cre* transgene under the control of the *Tie2* promoter and enhancer (Kisanuki et al., 2001). Homozygous deletion of endothelial *Erg* (*Tie2Cre-Erg*^*fl/fl*^; henceforth referred to as *Erg*^*cEC-KO*^) resulted in embryonic lethality between embryonic day (E)10.5 and E11.5, with no live offspring (Figure S1B). Analysis of the yolk sacs from *Erg*^*cEC-KO*^ embryos at E10.5 showed a significant reduction in perfused large vessels, consistent with defects in yolk sac vascular remodeling (Figures 1A, 5E, and 5F). Between E10.5 and E11.5, some mutant embryos appeared pale with no evidence of vessel blood flow, whereas others displayed hemorrhages and an enlarged pericardial cavity, suggesting defective heart function (Figures S1C and S1D). *Erg*^*cEC-KO*^ embryos were reduced in size compared to littermate controls (Figure 1B), with growth retardation clearly visible at E9.5 (Figure S1E). These results are in agreement with the phenotypes caused by global deletion of endothelial ERG isoforms (Vijayaraj et al., 2012). Endomucin staining of blood vessels in *Erg*^*cEC-KO*^ embryos at E10.5 revealed an immature disorganized vascular plexus, with significant disruption of the large vessels in the cranial vasculature (Figure 1B, panels a and b), altered development of the hyaloid vessels of the eye (Figure S1F), and irregular blind ending vessels in the head microvasculature (Figure S1G). Mutant embryos also exhibited disorganization of the intersomitic vessels in the trunk (Figure 1B, panel c). These data confirm that ERG is required for vascular development in mice.

To investigate the role of ERG in physiological and pathological postnatal angiogenesis, floxed *Erg* mice were bred with mice carrying tamoxifen-inducible Cre recombinase under the control of the *Cdh5* promoter (Cdh5(PAC)-iCreERT2) (Wang et al., 2010). Following tamoxifen administration, efficient Cre-recombinase deletion of *Erg* was confirmed by PCR in *Erg*^*fl/fl*^/Cdh5(PAC)-iCreERT2 mice (henceforth referred to as *Erg*^*iEC-KO*^) (Figure S1H). The reduction in ERG protein levels was demonstrated by western blotting and immunofluorescence microscopy (Figures S1I and S1J). In the retinal vasculature of littermate controls (*Erg*^*fl/fl*^), ERG was strongly expressed in endothelial cells (EC) from all regions of the vascular plexus, including tip and stalk cells, arteries, veins, and capillaries (Figure S1K), in line with previous studies (Korn et al., 2014). Deletion of endothelial *Erg* resulted in significant reduction of vascular coverage (Figure 1C) and density (Figure 1D) in the retinal plexus, and reduction in the number of vascular sprouts at the front (Figure 1E). Next, we investigated whether ERG is involved in pathological angiogenesis, using the B16F0 melanoma tumor model, which depends on angiogenesis for growth (Reynolds et al., 2002). At day 14, tumor size and microvessel density were significantly reduced in adult *Erg*^*iEC-KO*^ mice compared to controls (Figures 1F–1H). These studies confirm that ERG is required for postnatal angiogenesis and show that endothelial ERG is involved in tumor angiogenesis and tumor growth.

### ERG Controls Vascular Stability and Pericyte Coverage

**Figure 2:**
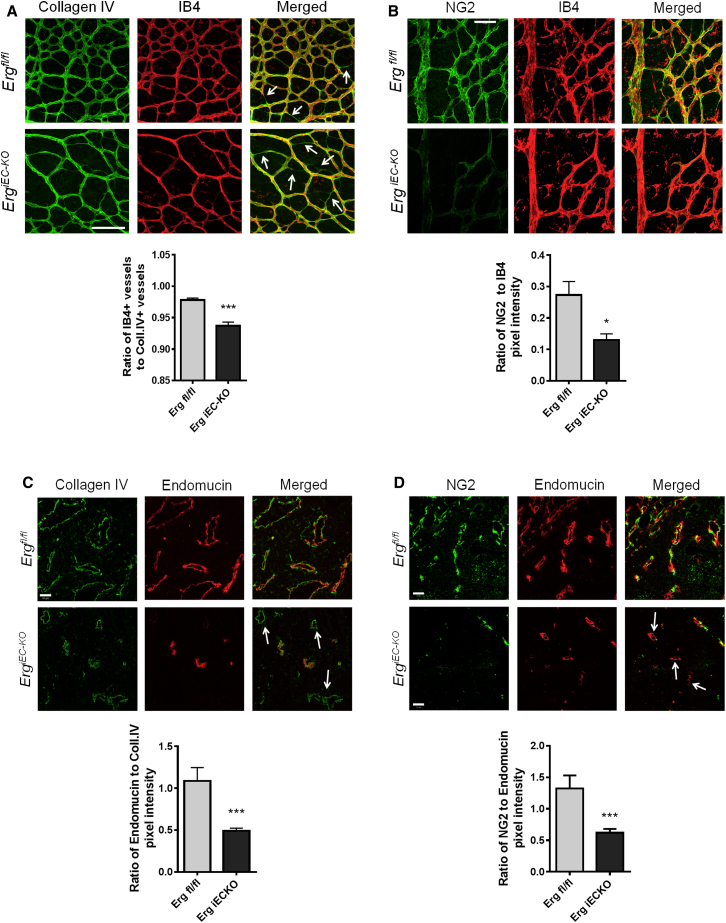
ERG Controls Vascular Remodeling (A) Collagen IV (green) and isolectin B4 (IB4, red) staining of *Erg*^*iEC-KO*^ and *Erg*^*fl/fl*^ P6 retinal vessels. Arrows show empty collagen IV sleeves, quantification of number of vessels, (n = 4). (B) NG2-positive pericytes (green) associated with isolectin B4 labeled retinal vessels (red) from *Erg*^*iEC-KO*^ and *Erg*^*fl/fl*^ mice; quantification of pixel intensity, (n = 4). (C) Sections from B16F0 tumors grown on adult *Erg*^*iEC-KO*^ and *Erg*^*fl/fl*^ mice were stained for collagen IV (green) and endomucin (red); quantification of pixel intensity, (n = 3). Arrows show empty collagen IV sleeves. (D) Tumor sections from *Erg*^*iEC-KO*^ and *Erg*^*fl/fl*^ mice were stained for NG2 (green) and endomucin (red); quantification of pixel intensity, (n = 3). Arrows show NG2-negative, endomucin-positive vessels. Scale bars, 100 μm (A), scale bars, 50 μm (B–D). All graphical data are ± SEM, ^∗^p < 0.05, and ^∗∗∗^p < 0.001. See also Figure S2.

Co-staining for isolectin B4 and the basement matrix component collagen IV showed a greater number of empty collagen IV sleeves in the capillary plexus (Figure 2A) and at the angiogenic front (Figure S2A) in retinas from *Erg*^*iEC-KO*^ mice compared to controls, indicating increased vessel regression. Pericyte recruitment, measured by staining with neuron-glial antigen 2 (NG2) and desmin, was significantly decreased along all vessels in the vascular plexus, including veins and arteries in *Erg*^*iEC-KO*^ mice (Figures 2B, S2B, and S2C). Similar signs of decreased vessel stability were observed in tumors grown in *Erg*^*iEC-KO*^ mice, with a marked increase in the number of empty collagen IV sleeves (Figure 2C) and a reduction in pericyte coverage of blood vessels (Figure 2D). These data suggest that ERG controls both physiological and pathological angiogenesis through pathways that promote vascular stability.

### ERG Controls β-Catenin Stability and Signaling through VE-Cadherin- and Wnt-Dependent Mechanisms

**Figure 3:**
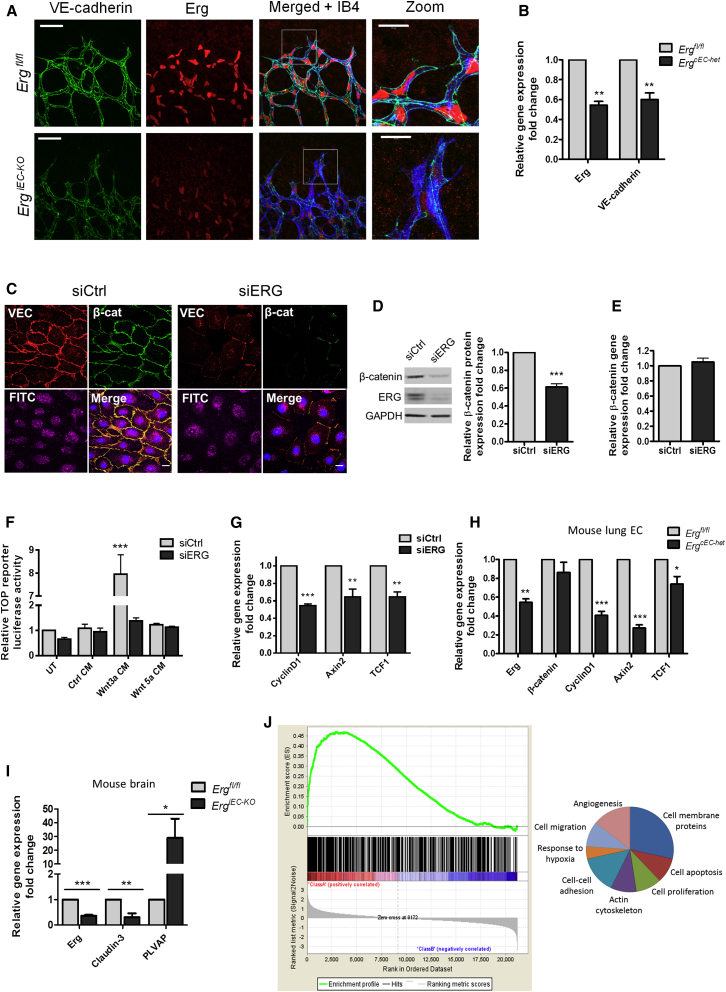
Endothelial Canonical Wnt Signaling and β-Catenin Stability Are Regulated by ERG (A) Staining for VE-cadherin (green), ERG (red), and isolectin B4 (IB4, blue) in *Erg*^*iEC-KO*^ and *Erg*^*fl/fl*^ P6 retinas. Scale bar, 50 μm; zoom, 20 μm. (B) Relative mRNA expression of Erg and VE-cadherin in primary *Erg*^*cEC-het*^ mouse lung EC compared to control (n = 6). (C) β-catenin (β-cat; green) and VE-cadherin (VEC; red) staining of FITC-conjugated siCtrl and siERG (FITC; purple) treated HUVEC (n = 3). Scale bar, 20 μm. (D and E) (D) Western blot and (E) qPCR analysis of β-catenin expression in control (siCtrl) and ERG-deficient (siERG) HUVEC (n = 4). (F) TCF reporter activity (TOP) in control and ERG-deficient cells treated with control (Ctrl), Wnt3a, or Wnt5a conditioned medium (CM); (n = 3). (G) qPCR of downstream β-catenin target gene expression in control and ERG-deficient HUVEC: Cyclin D1, Axin-2, and TCF-1 (n = 4). (H) mRNA expression of Erg, β-catenin, and its target genes Cyclin D1, Axin-2, and TCF-1 in primary *Erg*^*cEC-het*^ mouse lung EC compared to control (n = 6). (I) qPCR analysis of total brain mRNA from control and *Erg*^*iEC-KO*^ mice for Erg, Claudin-3, and PLVAP. (J) GSEA shows enrichment and significant correlation (normalized enrichment score, 2.46; p < 0.001) between genes downregulated in β-catenin siRNA-treated HPAEC (green curve) (Alastalo et al., 2011) and the ranked list of genes downregulated by ERG inhibition in HUVEC (Birdsey et al., 2012). Functional classification of the shared genes identified by GSEA was carried out using DAVID analysis (right). The functional categories shown displayed significant enrichment scores (p < 0.01). All graphical data are ± SEM, ^∗^p < 0.05, ^∗∗^p < 0.01, and ^∗∗∗^p < 0.001. See also Figure S3.

In vitro studies have shown that ERG is essential to maintain the integrity of endothelial junctions, by driving expression of VE-cadherin (Gory et al., 1998; Birdsey et al., 2008). A marked reduction in VE-cadherin expression and junctional localization was also observed in the retinal vasculature of *Erg*^*iEC-KO*^ mice (Figure 3A), demonstrating that loss of endothelial ERG leads to a disruption of cell-cell junctions in vivo. Isolated primary mouse lung EC from heterozygous *Tie2Cre-Erg*^*fl/+*^ mice (*Erg*^*cEC-het*^) (Figure S3A) showed an approximate 50% decrease in VE-cadherin expression (Figure 3B), as expected.

At the junctions, VE-cadherin binds β-catenin, protecting it from degradation; this interaction is required for the control of junction stabilization (Dejana, 2010). β-catenin plays a pivotal role in Wnt signaling (Goodwin and D’Amore, 2002); interestingly, transcription profiling in ERG-deficient human umbilical vein EC (HUVEC) identified several Wnt-related genes as candidate ERG targets (Birdsey et al., 2012) (Figure S3B). We therefore speculated that ERG might regulate β-catenin and Wnt signaling in EC.

Inhibition of ERG expression in HUVEC (Figures S3C and S3D) significantly reduced β-catenin junctional staining and protein expression (Figures 3C and 3D). ERG regulates β-catenin protein levels in confluent (Figure 3C) and subconfluent (Figure S3E) EC, suggesting that this pathway functions both in quiescent and angiogenic endothelium. However, β-catenin mRNA levels were unaffected by ERG inhibition in HUVEC (Figures 3E and S3F) or in primary mouse EC from *Erg*^*cEC-het*^ mice (Figure 3H). The decrease in β-catenin protein expression correlated with a decrease in canonical Wnt signaling: Wnt3a stimulation of β-catenin transcriptional activity was lost in ERG-deficient EC (Figure 3F). Moreover, expression levels of the Wnt target genes Cyclin D1, Axin-2, and TCF-1 (Shtutman et al., 1999; Roose et al., 1999; Jho et al., 2002) were decreased in ERG-deficient HUVEC (Figure 3G) and primary mouse EC from *Erg*^*cEC-het*^ mice (Figure 3H). Endothelial β-catenin signaling regulates blood brain barrier maintenance through concomitant activation of the tight junction molecule Claudin-3 and repression of plasmalemma vesicle-associated protein (PLVAP) (Liebner et al., 2008). In line with these findings, Claudin-3 expression was significantly downregulated, while PLVAP was strongly upregulated in *Erg*^*iEC-KO*^ mouse brains (Figure 3I). Together, these results indicate that ERG regulates canonical Wnt/β-catenin signaling in both human and mouse EC.

To confirm the relationship between ERG and β-catenin pathways, we used gene set enrichment analysis (GSEA) to compare the data set from transcriptome profiling of ERG-deficient HUVEC (Birdsey et al., 2012) with the data set from transcriptome analysis of human pulmonary artery EC (HPAEC) following β-catenin inhibition (Alastalo et al., 2011). GSEA showed a significant positive correlation between the genes regulated by ERG and β-catenin (Figure 3J, left). Interestingly, gene ontology analysis of shared genes identified a significant number of regulators of angiogenesis, cell adhesion, migration, and apoptosis (Figure 3J, right). These results suggest a strong relationship between these two pathways.

**Figure 4:**
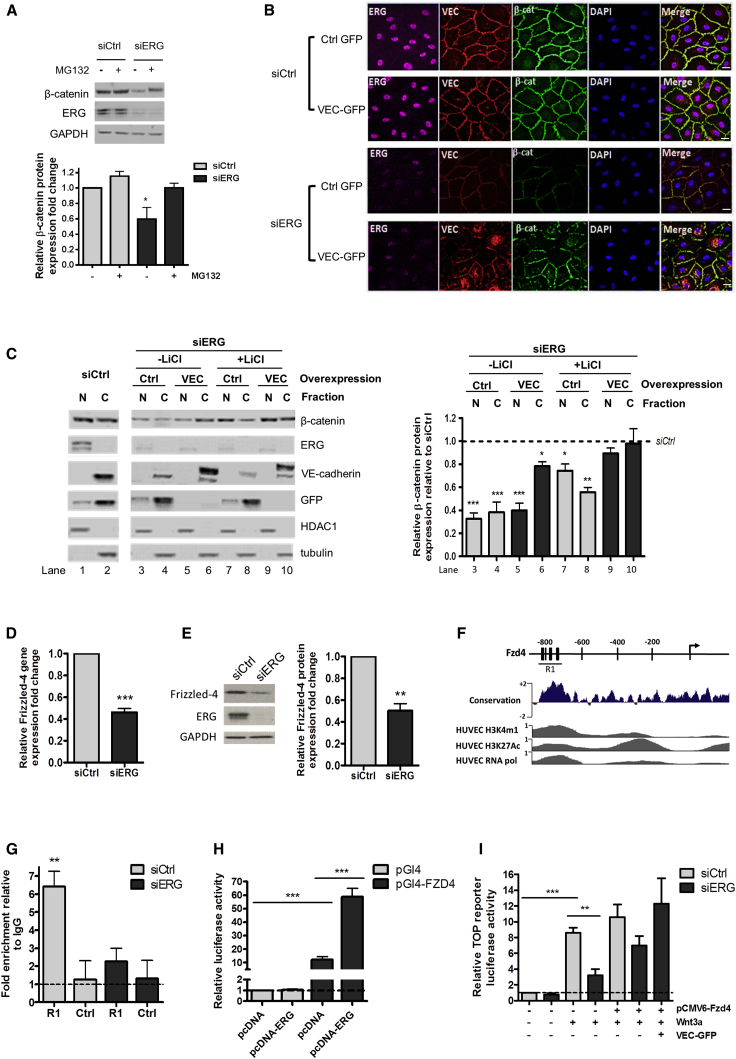
ERG Controls β-Catenin Stability through VE-Cadherin- and Wnt-Dependent Mechanisms (A) Western blot of β-catenin expression in control and ERG-deficient cells treated in presence or absence of MG132 (n = 4). (B) ERG (magenta), VEC (red), β-cat (green), and DAPI (blue) staining of control and ERG-deficient HUVEC transduced with GFP-tagged control (Ctrl-GFP) or VE-cadherin (VEC-GFP) adenovirus. Scale bar, 20 μm. (C) Western blot (left) and quantification (right) of β-catenin expression in nuclear/cytoplasmic fractions of ERG-deficient HUVEC transduced with GFP or VEC-GFP adenovirus in presence or absence of LiCl. For normalization, tubulin was used as a cytoplasmic control and HDAC1 as a nuclear marker (n = 3). (D and E) (D) qPCR and (E) western blot analysis of Fzd4 expression in control and ERG-deficient cells (n = 3). (F) There are three putative ERG binding sites (black bars) located within the Fzd4 locus upstream of the transcription start site (arrow); sequence conservation between 100 vertebrates is shown across this region. ENCODE ChIP-seq data profiles for H3K4me1, H3K27Ac, and RNA polymerase II indicate open chromatin and active transcription. Location of qPCR amplicon covering region R1 is indicated. (G) ChIP-qPCR using primers to region R1 on ERG-bound chromatin from HUVEC treated with siCtrl or siERG. Primers for a downstream region within the Fzd4 gene were used as a negative control. Data are shown as fold change over IgG (n = 3). (H) Luciferase reporter assay, an ERG cDNA expression plasmid (pcDNA-ERG), or an empty expression plasmid (pcDNA) were cotransfected with a Fzd4 promoter-luciferase construct (pGl4-Fzd4) in HUVEC and luciferase activity was measured. Values are represented as the fold change in relative luciferase activity over the empty pGL4 vector alone. (I) TCF reporter (TOP) activity in control and ERG-deficient HUVEC treated with rWnt3a. Cells were transfected with control pCMV6 or pCMV6-Fzd4 plasmids and transduced with VEC-GFP adenovirus (n = 3). All graphical data are ± SEM, ^∗^p < 0.05, ^∗∗^p < 0.01, and ^∗∗∗^p < 0.001. See also Figure S4.

Since ERG inhibition decreases β-catenin protein, but not mRNA levels, we tested whether ERG regulates β-catenin degradation. β-catenin protein expression was restored in ERG-deficient EC in the presence of the proteosomal degradation inhibitor MG132 (Figure 4A). We investigated whether ERG controls β-catenin stability through VE-cadherin. In ERG-deficient HUVEC, GFP-tagged VE-cadherin overexpression (Figure S4A) partially restored junctional β-catenin protein levels (Figures 4B and 4C, lane 6). However, cellular fractionation studies showed that ERG also controls the nuclear pool of β-catenin (Figure 4C, lane 3), which was not corrected by VE-cadherin overexpression (Figure 4C, lane 5). This suggests that ERG controls β-catenin also through a Wnt signaling-dependent, VE-cadherin-independent pathway. Activation of Wnt signaling by lithium chloride (LiCl) inhibits GSK3β, and thus degradation of cytoplasmic β-catenin, allowing its nuclear translocation (Stambolic et al., 1996). LiCl was able to partially normalize β-catenin nuclear levels in ERG-deficient EC (Figure 4C, lane 7). Finally, combined Wnt signaling activation (through LiCl) and VE-cadherin overexpression were able to rescue both nuclear and junctional β-catenin pools in ERG-deficient EC (Figure 4C, lanes 9 and 10). These results demonstrate that the balance between VE-cadherin-dependent and Wnt signaling-dependent pathways, which modulates canonical Wnt/β-catenin signals in EC, is controlled by the transcription factor ERG.

### Expression of the Wnt Receptor Frizzled-4 Is Regulated by ERG

Wnt ligands bind to receptors of the Fzd family to inhibit the β-catenin degradation complex and activate Wnt signaling (Goodwin and D’Amore, 2002). LiCl treatment was able to partially stabilize β-catenin expression in ERG-deficient EC (Figure 4C, lanes 7 and 8). However, the upstream ligand Wnt3a was unable to do so (Figure S4B), suggesting a receptor-mediated defect upstream of the degradation complex. Wnt3a interacts with the Frizzled-4 (Fzd4) receptor (Reis and Liebner, 2013), which is highly expressed in cultured EC (Goodwin et al., 2006). Fzd4 was identified as a putative ERG target by transcriptome analysis in HUVEC (Birdsey et al., 2012). We confirmed that Fzd4 mRNA (Figure 4D) and protein levels (Figure 4E) were significantly decreased in ERG-deficient HUVEC. Consistently, Fzd4 expression was decreased in mouse EC isolated from *Erg*^*cEC-het*^ mice (Figure S4C).

Comparative genomic analysis of the Fzd4 promoter revealed the presence of three highly conserved ERG DNA binding motifs in the 800 base pair (bp) region upstream of the Fzd4 transcription start site (Figures 4F and S4D). Analysis of the Encyclopedia of DNA Elements (ENCODE) chromatin immunoprecipitation sequencing (ChIP-seq) data (Birney et al., 2007) for histone marks H3K4me1 and H3K27Ac and RNA polymerase II occupancy, markers of active promoters, show that the location of these marks correlates with the position of the ERG binding motifs (Figure 4F). ChIP-quantitative (q)PCR demonstrated that ERG interacts directly with the human Fzd4 promoter (Figure 4G); specificity of the interaction was confirmed in ERG-deficient EC (Figure 4G). ERG overexpression resulted in a 6-fold transactivation of a Fzd4 promoter luciferase construct in HUVEC (Figure 4H). Finally, Fzd4 overexpression in ERG-deficient EC was able to partially rescue Wnt3a activation of β-catenin transcriptional activity (Figure 4I). These data demonstrate that ERG controls transcription of the Fzd4 receptor in EC and point to a molecular mechanism for the VE-cadherin-independent control of Wnt signaling by ERG.

### ERG Controls Angiogenesis through Wnt Signaling

**Figure 5:**
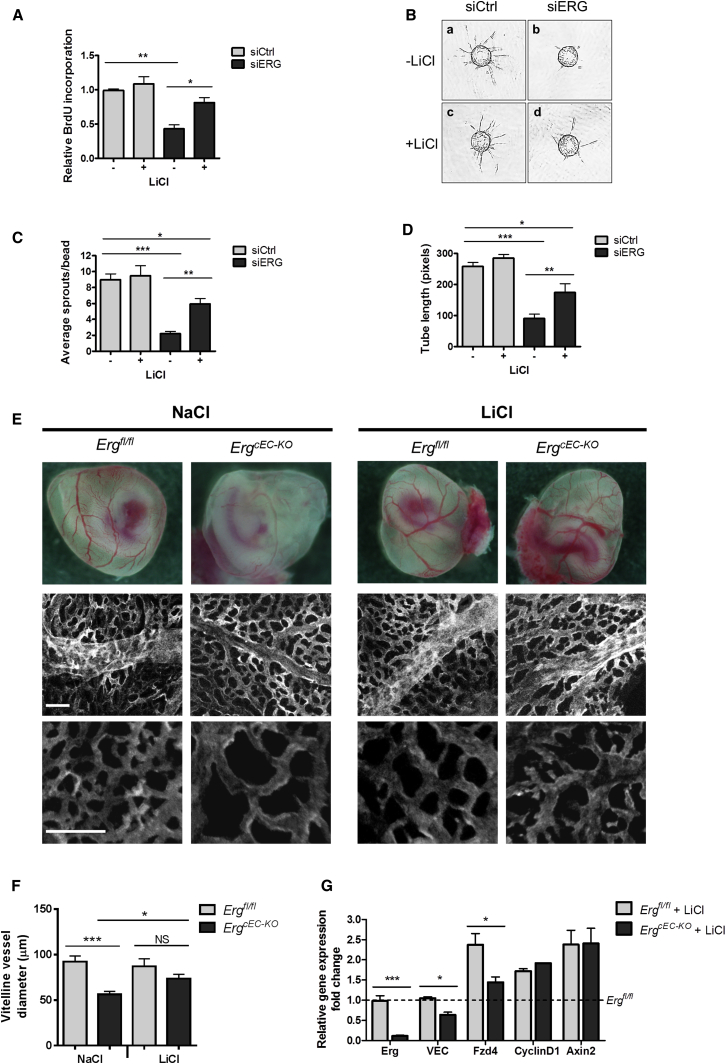
ERG Regulates Angiogenesis through Wnt/β-Catenin Signaling (A) In vitro Brdu incorporation in control and ERG-deficient HUVEC treated in presence or absence of LiCl (n = 4). (B–D) (B) Representative images of EC sprouts on fibrin gel beads using siCtrl or siERG-treated HUVEC in the presence or absence of LiCl; (C) quantification of numbers of sprouts; and (D) tube length (n = 20). (E) (Top) Representative whole mount images of E10.5 *Erg*^*fl/fl*^ and *Erg*^*cEC-KO*^ embryo yolk sacs from pregnant female mice treated with either NaCl (left) or LiCl (right) at E8.5 and E9.5. Scale bar, 1 mm (n = 5). (Middle and bottom panels) Endomucin staining of yolk sac vasculature; scale bar, 100 μm. (F) Quantification of yolk sac vitelline vessel diameter. (G) qPCR analysis of LiCl-treated *Erg*^*fl/fl*^ and *Erg*^*cEC-KO*^ embryo yolk sacs. Data are expressed as fold change versus NaCl-treated *Erg*^*fl/fl*^ and are ± SEM from at least three mice per group. All graphical data are ± SEM, ^∗^p < 0.05, ^∗∗^p < 0.01, and ^∗∗∗^p < 0.001. See also Figure S5.

Wnt/β-catenin signaling can promote EC proliferation (Masckauchan et al., 2005) and induce cell cycle progression through transcriptional activation of Cyclin D1 (Shtutman et al., 1999). Therefore, we tested whether ERG may also control EC proliferation through Wnt signaling. As shown in Figure 5A, inhibition of ERG expression by siRNA decreased HUVEC proliferation; LiCl, which prevents β-catenin degradation, rescued proliferation of ERG-deficient HUVEC. These results indicate that ERG controls endothelial proliferation through the Wnt/β-catenin pathway. We have previously shown that ERG deficiency causes increased EC apoptosis (Birdsey et al., 2008). Combination of VE-cadherin overexpression and LiCl treatment could completely prevent cell death in ERG-deficient cells, indicating that ERG controls EC survival through Wnt/β-catenin signaling (Figure S5A).

To test the functional relevance of Wnt signaling in ERG-dependent angiogenesis, we used an in vitro sprouting assay (Nakatsu et al., 2007). ERG-deficient HUVEC formed markedly decreased numbers of significantly shorter sprouts (Figures 5B, panel b, 5C, and 5D). However, pretreatment of ERG-deficient cells with LiCl to inhibit β-catenin degradation was able to partially restore normal sprouting behavior of HUVEC (Figure 5B, panel d), by rescuing the number (Figure 5C) and length (Figure 5D) of the sprouts. These results suggest that Wnt/β-catenin signaling is required for ERG to control sprout formation during angiogenesis.

To confirm that ERG controls angiogenesis and vascular development in a Wnt/β-catenin-dependent manner in vivo, we carried out a rescue experiment by pharmacological stabilization of Wnt/β-catenin signaling (Griffin et al., 2011). Light microscopy examination of the yolk sacs from NaCl (control) and LiCl treated mice revealed a dramatic increase in perfused vessels in the yolk sacs of *Erg*^*cEC-KO*^ mutants following LiCl treatment (Figure 5E). Endomucin staining revealed disrupted vessel morphology in the yolk sacs from NaCl-treated *Erg*^*cEC-KO*^ embryos, with reduced microvasculature branching and decreased diameter of the larger vitelline vessels (Figures 5E and 5F). LiCl treatment of *Erg*^*cEC-KO*^ mutants resulted in significant increase in vitelline vessel diameter and in the remodeling of the microvascular plexus (Figures 5E and 5F), in line with the increase in perfusion.

Wnt targets CyclinD1 and Axin2, previously shown to be decreased in ERG-deficient endothelium (see Figures 3G and 3H), were significantly decreased in NaCl-treated *Erg*^*cEC-KO*^ yolk sacs compared to controls (Figure S5B). LiCl-treatment of *Erg*^*cEC-KO*^ yolk sacs normalized Cyclin D1 and Axin2 levels to those observed in LiCl-treated control embryos (Figure 5G). Interestingly, ERG targets VE-cadherin and Fzd4 were not normalized, in line with the direct transcriptional role of ERG in their regulation.

These results demonstrate that endothelial ERG controls embryonic vascular development and angiogenesis through the Wnt/β-catenin signaling pathway.

### ERG Overexpression Stabilizes VEGF-Induced Blood Vessels and Promotes Angiogenesis In Vivo

The data presented so far show that the transcription factor ERG controls angiogenesis through pathways mediating vascular stability and growth. The importance of the coordinated regulation of these pathways is highlighted by the variable and disappointing results of clinical trials for therapeutic angiogenesis in ischemic diseases, using the proangiogenic growth factor VEGF. VEGF has been shown to induce the formation of unstable and highly permeable vessels in vivo (Reginato et al., 2011), giving rise to local edema and inefficient tissue perfusion. Therefore, we investigated the ability of ERG to stabilize new vessels induced by VEGF in vivo. C57BL/6 mice received a subcutaneous injection of Matrigel supplemented with VEGF-A_165_ and ERG (Ad.ERG) or Lacz (Ad.Lacz) adenovirus; basic fibroblast growth factor (bFGF), which can induce stable new vessels in this model (Bussolati et al., 2004), was used as control. Immunofluorescence staining for the adenovirus hexon coat protein showed localization of the adenovirus to endomucin-positive neovessels in the Matrigel plugs (Figure S6A). In addition, qPCR analysis confirmed significant expression of V5-tagged ERG in Matrigel samples treated with Ad.ERG compared to Ad.Lacz control (Figure S6B).

**Figure 6:**
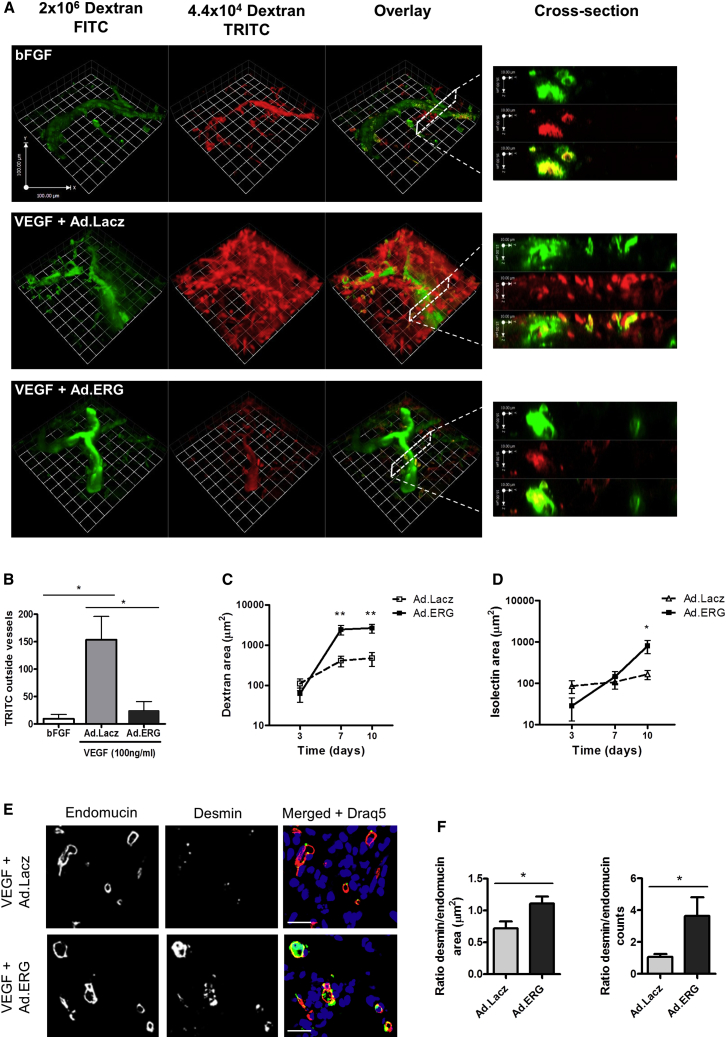
ERG Stabilizes Angiogenesis In Vivo Matrigel containing bFGF or VEGF with adenovirus expressing either Lacz (Ad.Lacz) or ERG (Ad.ERG) was injected into C57BL6 mice. There were two labeled dextran molecules of different molecular weights, 2×10^6^MW (FITC, green) and 4.4×10^4^ MW (TRITC, red), that were injected intravenously 15 min prior to harvesting plugs. (A) 3D rendering of confocal microscopy images of whole-mount Matrigel plugs perfused with the dextran tracers. Cross sectioning through neovessels (right) shows localization of the tracers. (B) Vessel permeability was quantified by measuring the amount of dextran-TRITC present outside of the dextran-FITC positive vessels, arbitrary units (n = 3). (C) Perfused vessels were quantified by measuring the area of dextran-FITC within the Matrigel plug after 3, 7, and 10 days (n = 4). (D) Vessel density was quantified by measuring the area of isolectin B4 within the Matrigel plug after 3, 7, and 10 days (n = 4). (E) Endomucin (red), desmin-positive pericytes (green), and Draq5 (blue) staining of cryosections from Matrigel plugs implanted for 7 days; scale bar, 20 μm. (F) Quantification of pericyte coverage, pixel intensity (n = 8). All graphical data are ± SEM, ^∗^p < 0.05, and ^∗∗^p < 0.01. See also Figure S6.

To evaluate the stability of the new vessels, vascular permeability was measured using two different sized dextran tracers. In the presence of bFGF (Figure 6A, top panel), the lower molecular weight tetramethylrhodamine (TRITC)-dextran was fully contained within the vascular structures and colocalized with the larger molecular weight Fluorescein isothiocyanate (FITC)-tracer, confirming that bFGF induces the formation of stable, nonleaky vessels. Plugs containing VEGF-A_165_ and Ad.Lacz revealed a less organized vascular network with the smaller molecular weight dextran dispersed both inside and outside of the vessels (Figure 6A, middle panel, and Movie S1A). Interestingly, overexpression of ERG in the presence of VEGF-A_165_ resulted in reduced diffusion of the smaller molecular weight dextran (Figure 6A, bottom panel, and Movie S1B), indicating a more stable, less permeable vasculature. Quantification of the net amount of extravasated TRITC-dextran tracer shows that Ad.ERG caused an approximate 4-fold reduction in tracer extravasation in VEGF-A_165_-induced new vessels compared to Ad.Lacz control (Figure 6B). Consistently, quantification of FITC-dextran area showed that ERG overexpression in the presence of VEGF-A_165_ resulted in an increase in perfused vessels within the Matrigel plug after 7 and 10 days, compared to control (Figures 6C and S6C). ERG overexpression also resulted in a significant increase in the number of new vessels within the Matrigel plug; however this difference was observed only at the later time point (Figure 6D), suggesting that the increase in blood vessel number is secondary mainly to stabilization of VEGF-induced angiogenesis. These results confirm that ERG promotes stabilization of VEGF-induced angiogenesis in vivo.

Pericyte recruitment is a critical step in vascular stability and maturation, and lack of pericytes has been shown to cause increased permeability (Hellström et al., 2001). Since pericyte recruitment was decreased in two models of angiogenesis in the *Erg*^*iEC-KO*^ mice (see Figure 2), we investigated whether ERG overexpression could increase the recruitment of vascular pericytes in the in vivo Matrigel plug model. Indeed, pericyte recruitment as measured by desmin staining was increased in the Ad.ERG-treated plugs compared to controls (Figures 6E and 6F). These results suggest that ERG may promote stabilization of angiogenesis also through control of pericyte recruitment.

## Discussion

Over the last decade, major progress has been made in understanding the molecular mechanisms that regulate angiogenesis. However, the pathways that control vessel stability are less well characterized. In this study, we identify a transcriptional program regulated by ERG that controls vascular stability and growth through the Wnt/β-catenin pathway, in both a physiological and pathological context.

We show that constitutive deletion of endothelial ERG in the mouse embryo causes embryonic lethality with severe vascular disruption. These observations are in line with a previous report where global deletion of a subset of endothelial ERG isoforms resulted in vascular defects and lethality between E10.5 and E11.5 (Vijayaraj et al., 2012). Instead of a strategy based on a posteriori knowledge of ERG isoform expression, the *Cre/LoxP* system allowed us to delete all endothelial isoforms of *Erg*, by targeting exon 6, which encodes a region of the protein present in all isoforms. A previous transgenic model, where ERG’s function was disrupted by a mutation in the DNA binding ETS domain (*Erg*^Mld2/Mld2^), caused embryonic lethality at a later stage (E13.5) (Loughran et al., 2008) and did not appear to display early vascular defects, suggesting that ERG’s functions in the vasculature are not exclusively mediated by its DNA binding activity.

Using the inducible endothelial specific Cdh5(PAC)-iCreERT2 line, we show that ERG is required for angiogenesis in the developing retina of newborn mice and for tumor blood vessel growth in adult mice. ERG deficiency results in vessel regression and reduced pericyte recruitment, demonstrating that ERG controls vascular stability. Interestingly, ERG overexpression in the in vivo Matrigel plug model resulted in increased pericyte recruitment to vessels. This suggests that ERG may promote stabilization of angiogenesis in part through control of pericyte recruitment. A recent paper has described a role for ETS factors (including ERG) in arterial specification and reported increased ERG expression in arterial-derived EC in vitro (Wythe et al., 2013). However, in the mouse retinal vasculature, ERG was strongly expressed in all EC with no detectable difference between arteries and veins.

The in vivo developmental vascular defects in the *Erg*^*cEC-KO*^ embryos are reminiscent of those associated with deletion of endothelial β-catenin. Endothelial deletion of ERG causes embryonic lethality earlier than the E12.5 reported for endothelial deletion of β-catenin (Cattelino et al., 2003). This study proposed that EC might not require β-catenin for early vascular development, but rather for maintenance of vascular integrity and vascular patterning at later stages. ERG’s regulation of other genes involved in earlier stages of vascular development, such as VE-cadherin, may be partly accountable for this difference in phenotypes. Constitutive endothelial deletion of ERG also causes diffuse hemorrhages and defects in vascular remodeling, similar to those observed in the β-catenin deficient embryos. In both lines, vitelline vessels of the yolk sac are significantly smaller in diameter; however, unlike *Erg*^*cEC-KO*^ embryos, endothelial-specific loss of β-catenin does not affect vessel formation in the head, but causes hyperbranching of intersomitic vessels (Corada et al., 2010). Thus, embryonic mouse phenotypes of ERG versus β-catenin endothelial deletion show similarities, but not complete overlap, as expected, given the complex role of ERG as a transcriptional regulator of multiple vascular pathways. Crucially, the yolk sac vascular defects in the *Erg*^*cEC-KO*^ and expression of Wnt targets were rescued by in vivo treatment with LiCl. Although we cannot completely rule out non-EC effects of LiCl, these experiments clearly demonstrate that ERG controls vascular development through Wnt signaling.

Interestingly, similar angiogenic defects are observed in the retinas from *Erg*^*iEC-KO*^ and from the reported endothelial-specific β-catenin and Fzd4 knockout mice (Xu et al., 2004; Ye et al., 2009; Phng et al., 2009; Corada et al., 2010). Whether ERG is implicated in human ocular diseases, including Norrie disease and familial exudative vitreoretinopathy, which are associated with Fzd4 and its ligand Norrin (Xu et al., 2004), remains to be established. In line with our data, a link between Fzd4 and ERG has been previously observed in prostate cancer (Gupta et al., 2010). Interestingly, our results show that ERG deficiency results in about 50% reduction in Fzd4 protein, but completely abrogates Wnt luciferase reporter activity in response to Wnt3a. This suggests that ERG’s control of other nodes in this pathway, including repression of the Wnt inhibitor DACT1 (Zhang et al., 2006) and activation of the transcription factor TCF4 (Wang et al., 2002), may be important.

Canonical Wnt signaling promotes EC survival, junction stabilization, proliferation, and pericyte recruitment and is essential for vessel stability (Cattelino et al., 2003; Phng et al., 2009; reviewed in Franco et al., 2009; Dejana, 2010). In this study, we establish ERG as a regulator of canonical Wnt/β-catenin signaling, and therefore identify a connection between two key transcriptional regulators essential for EC function. We show that ERG controls cell survival, proliferation, angiogenesis, and vessel stability through β-catenin. Whether ERG controls pericyte recruitment via Wnt signaling remains to be elucidated; preliminary evidence suggests that ERG regulates expression of the junction molecule and β-catenin transcriptional target N-cadherin (data not shown), which plays a crucial role in pericyte attachment during vessel formation (Giampietro et al., 2012; Gerhardt et al., 2000). Our data show that ERG controls Wnt/β-catenin levels and signaling through VE-cadherin-dependent and -independent pathways both in confluent, quiescent monolayers and in subconfluent, proliferating cells. The balance between VE-cadherin and Wnt-dependent signals controls β-catenin cellular localization and activity. It has been suggested that β-catenin could function to increase cell plasticity and sensitivity to extracellular signals (Franco et al., 2009). Transcriptional activity of ERG itself has been shown to be modulated by extracellular signals (Wythe et al., 2013). Thus, we propose that in EC, ERG is required to maintain homeostatic levels of β-catenin protein, the output of which can be modulated according to the growth and survival signals it encounters, providing the balance between proliferation and stability required in a nascent blood vessel.

Dysregulation of the Wnt/β-catenin signaling pathway is frequently observed in many types of cancer. Constitutive Wnt signaling activation caused by mutations in β-catenin or genes that control β-catenin stability has been associated with aberrant cell proliferation and subsequent cancer progression (reviewed in Giles et al., 2003). Wnt signaling has been shown to be a critical mediator of ERG-induced oncogenesis in several types of cancer, where aberrant ERG overexpression is a marker of aggressive malignancy and associated with increased proliferation (Gupta et al., 2010; Wu et al., 2013; Li et al., 2011; Mochmann et al., 2011). This is in contrast with its role in healthy endothelium, where ERG promotes homeostasis and stability. The reasons for this discrepancy are unknown and may be linked to the lack of balance between growth and survival signals, due to disrupted cell-cell signaling in malignant cells, thus driving the cells to a proliferative fate. Thus, strategies to control ERG’s activity in malignant cells through cell-cell adhesion signals might be worthy of investigation.

Finally, in this study, we explore the potential for ERG in promoting vascular stability during VEGF-induced angiogenesis. Numerous studies have shown that the new vasculature induced by VEGF in vivo, to promote revascularization in ischemic diseases (Zachary and Morgan, 2011), can be dysfunctional due to vascular instability and increased permeability (Reginato et al., 2011). Here, we show that overexpression of ERG can reduce permeability and promote VEGF-induced angiogenesis in vivo. Combined with the homeostatic and anti-inflammatory role of ERG (Sperone et al., 2011; Yuan et al., 2009), these results establish the ERG pathway as a potential target to promote vascular quiescence and stability.

## Experimental Procedures

Detailed methods are available in the Supplemental Experimental Procedures.

### Mice and Breeding

Generation of *Erg* floxed mice was carried out by genOway. LoxP sequences were inserted around exon 6. Deletion of this exon leads to a frameshift mutation resulting in a premature stop codon in exon 7. *Erg*^*fl/fl*^ mice were crossed with the following *Cre* transgenic deleter lines: *Cdh5(PAC)-CreERT2* (Wang et al., 2010) and *Tie2-Cre* (Kisanuki et al., 2001). All experiments were conducted in accordance with the Animals (Scientific Procedures) Act of 1986.

### Postnatal Retinal Angiogenesis

Mice were administered Tamoxifen (50 μg per mouse; Sigma) by intraperitoneal injection (IP) at postnatal (P) day 1, P2 and P3. Retinas were collected at P6 and processed as described (Pitulescu et al., 2010).

### Syngeneic Tumor Experiments

Mouse melanoma B16F0 tumors were grown in tamoxifen-treated adult *Erg*^*fl/fl*^ and *Erg*^*iEC-KO*^ mice as described (Reynolds et al., 2002).

### In Vivo LiCl Treatment

LiCl or NaCl (400 mg/kg, dissolved in water) was injected IP into pregnant female mice at E8.5 and E9.5. Embryos were harvested at E10.5 and yolk sac vasculature was analyzed by light microscopy and immunostaining.

### In Vivo Matrigel Angiogenesis Assay

C57BL/6 mice received a subcutaneous injection of Matrigel (BD Biosciences), as described (Birdsey et al., 2008). Matrigel was supplemented with 80 nanogram (ng)/ml bFGF (R&D Systems), or with 100 ng/ml murine VEGF-A_165_ (Peprotech) containing 10^9^ plaque-forming unit adenovirus expressing either Lacz or ERG. After 3, 7, or 10 days, 100 μl of a 1:1 mixture of 10 mg/ml Dextran:FITC (2×10^6^ MW) and Dextran:TRITC (4.4×10^4^ MW) was injected intravenously 15 min prior to harvesting plugs. Plugs were imaged whole-mount using confocal microscopy. Volocity software (Perkin Elmer) was used to reconstruct 3D images of the vessels from serial Z-sections. The extent of TRITC-dextran tracer extravasation was quantified by subtracting the signal corresponding to the FITC-dextran tracer (intravascular) from the signal corresponding to the TRITC-dextran tracer (intravascular + extravascular).

### Isolation of Mouse Lung Endothelial Cells

Primary mouse lung EC were isolated from control *Erg*^*fl/fl*^ and *Erg*^*cEC-het*^ mice as described (Reynolds et al., 2002). Rat APC-CD31, anti-ICAM-2, and anti-rat PE antibodies (all BD Biosciences) were used to assess the EC purity by flow cytometric analysis using a Cyan flow cytometer (Beckman Coulter).

### Cell Culture

Primary HUVEC were harvested from umbilical cords (Birdsey et al., 2008). Human *ERG* expression was inhibited using either GeneBloc antisense oligonucleotides (Silence Therapeutics) (McLaughlin et al., 2001) or siRNA against *ERG* (Hs_ERG_7); both denoted as siERG in the text. Control GeneBloc antisense or AllStars Negative Control siRNA (QIAGEN) are denoted as siCtrl.

### ChIP-qPCR

ChIP was performed using ChIP-IT express (Active Motif) as previously described (Birdsey et al., 2012).

### Plasmid Transfections and Reporter Assays

For Fzd4 transactivation assays, a 1,010-bp region of the Fzd4 promoter (SwitchGear, Active Motif) was cloned into the pGL4 Luciferase Reporter Vector (Promega). TCF reporter constructs TOPFLASH and FOPFLASH were used to measure the transcriptional activity of β-catenin/TCF (Korinek et al., 1997). In some experiments, cells were cotransfected with a pCMV6-Fzd4 expression construct (Origene). Reporter assays were performed using the Dual-Luciferase Reporter Assay System (Promega).

### BrdU In Vitro Proliferation Assay

Cell proliferation was determined in vitro using a BrdU proliferation ELISA kit (Roche) according to the manufacturer’s instructions.

### Fibrin Gel Bead Assay

The 3D in vitro model of angiogenesis was performed as described previously (Nakatsu et al., 2007).

### Statistical Analysis

Values are presented as means ± SEM. Statistical significance was determined by using unpaired two-tailed Student’s t test. Differences were considered significant with a p value < 0.05.

## Author Contributions

G.M.B. designed, carried out, and supervised in vivo and in vitro experiments, analyzed and interpreted results, and wrote the manuscript. A.V.S. designed and carried out in vitro experiments, analyzed, interpreted, and conceptualized results, and wrote the manuscript. N.D. designed and performed in vivo experiments, analyzed, and interpreted results. B.G. provided advice on bioinformatic analysis and interpretation and contributed to scientific discussion; Y.Y. performed bioinformatic analysis and interpretation; and L.R. and K.H.D. performed and supervised the tumor angiogenesis experiments, analyzed results, and contributed to scientific discussion. L.O.A. and S.T.K. performed experiments and analyzed results. I.M.A. provided advice on retina isolation and optimized ERG retinal staining; E.D. provided reagents and contributed to scientific discussion; J.C.M. contributed to scientific discussion; H.G. provided advice and contributed to scientific discussion; R.H.A. assisted in the design of the transgenic mice, provided reagents, advice, and contributed to scientific discussion; and A.M.R. provided funding, conceived, designed, and supervised the study, interpreted results, and wrote the manuscript.

## Supplemental Information

Document S1. Supplemental Experimental Procedures and Figures S1–S6

Movie S1. 3D Reconstruction of Neovessels inside Matrigel Plugs Supplemented with VEGF and Adenovirus Expressing Lacz, Related to Figure 6 Perfused vessels are labeled with FITC-dextran (green) and vessel leakage is visualized with TRITC-dextran (red).

Movie S2. 3D Reconstruction of Neovessels inside Matrigel Plugs Supplemented with VEGF and Adenovirus Expressing ERG, Related to Figure 6 Perfused vessels are labeled with FITC-dextran (green) and vessel leakage is visualized with TRITC-dextran (red).

Document S2. Article plus Supplemental Information
