# Supplementary material for: The Endothelial Transcription Factor ERG Promotes Vascular Stability and Growth through Wnt/β-Catenin Signaling
Source: Dev Cell. 2015 Jan 12;32(1):82–96. doi: 10.1016/j.devcel.2014.11.016 (PMC4292982; doi:10.1016/j.devcel.2014.11.016)
Supplement: Document S1. Supplemental Experimental Procedures and Figures S1–S6 [file mmc1.pdf]

Developmental Cell, Volume *32*
Supplemental Information

The Endothelial Transcription Factor ERG Promotes Vascular Stability and Growth through Wnt/β-Catenin Signaling

Graeme M. Birdsey, Aarti V. Shah, Neil Dufton, Louise E. Reynolds, Lourdes Osuna Almagro, Youwen Yang, Irene M. Aspalter, Samia T. Khan, Justin C. Mason, Elisabetta Dejana, Berthold Göttgens, Kairbaan Hodivala-Dilke, Holger Gerhardt, Ralf H. Adams, and Anna M. Randi

Figure S1

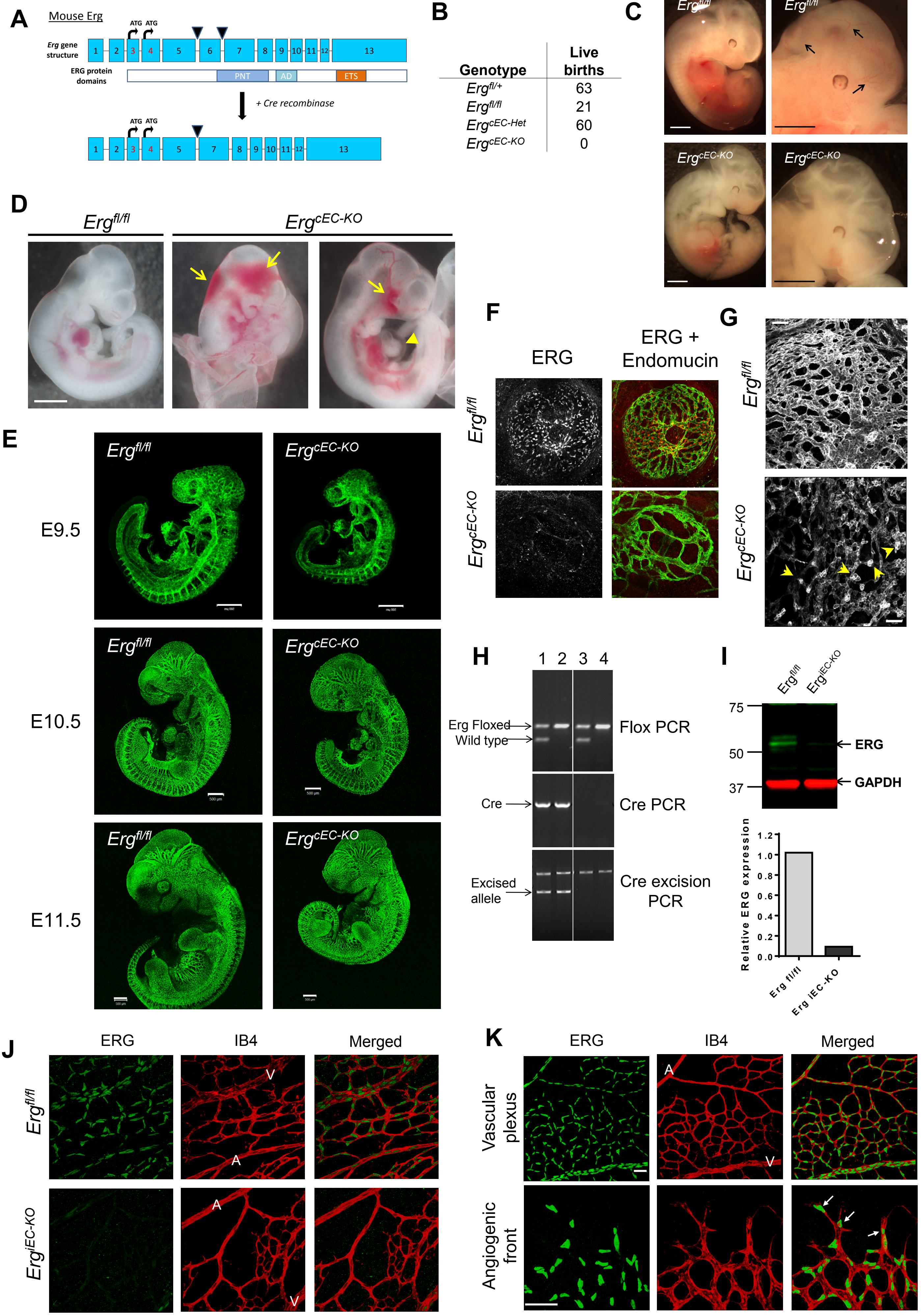
A
Mouse Erg
ATG ATG
Erg gene structure
1 2 3 4 5 6 7 8 9 10 11 12 13
ERG protein domains
PNT
AD
ETS
+ Cre recombinase
1 2 3 4 5 7 8 9 10 11 12 13
B
Genotype | Live births
Erg fl/+ | 63
Erg fl/fl | 21
Erg cEC-Het | 60
Erg cEC-KO | 0
C
Erg fl/fl
Erg cEC-KO
D
Erg fl/fl
Erg cEC-KO
E
E9.5
E10.5
E11.5
Erg fl/fl
Erg cEC-KO
F
ERG
ERG + Endomucin
Erg fl/fl
Erg cEC-KO
G
Erg fl/fl
Erg cEC-KO
H
1 2 3 4
Erg Floxed
Wild type
Flox PCR
Cre
Cre PCR
Excised allele
Cre excision PCR
I
Erg fl/fl
Erg iEC-KO
75
50
37
ERG
GAPDH
Relative ERG expression
1.2
1.0
0.8
0.6
0.4
0.2
0.0
Erg fl/fl
Erg iEC-KO
J
ERG
IB4
Merged
Erg fl/fl
Erg iEC-KO
A
V
K
ERG
IB4
Merged
Vascular plexus
Angiogenic front
A
V

Figure S1, Related to Figure 1

Generation of *Erg* floxed mouse; characterization of vascular phenotypes in *Tie2Cre-Erg*$^{fl/fl}$ (*Erg*$^{cEC\text{-}KO}$) and *Erg*$^{fl/fl}$/Cdh5(PAC)-iCre^{ER}T2 (*Erg*$^{iEC\text{-}KO}$) mice

(**A**) Schematic structure of the murine floxed *Erg* gene. The two translation start sites within the alternatively spliced exons 3 and 4 are shown (ATG). The location of the loxP sites flanking exon 6 are indicated (black arrowheads). The predicted ERG protein domains are indicated, showing the approximate location of the pointed domain (PNT), alternative domain (AD) and ETS DNA-binding domain (ETS). (**B**) Genotype frequencies of live births from *Erg* floxed mice, obtained from 8 breeding females. (**C**) Whole mount images of *Erg*$^{fl/fl}$ and *Erg*$^{cEC\text{-}KO}$ embryos. Arrows indicate perfused vessels in the head. Scale bar, 1 mm (**D**) Morphology of *Erg*$^{fl/fl}$ embryos (left) compared to *Erg*$^{cEC\text{-}KO}$ (middle and right) embryos, which display hemorrhaging in the head (arrows), and fluid accumulation around the pericardial cavity (arrowhead). Scale bar, 1 mm. (**E**) Confocal microscopy showing endomucin-staining of blood vessels in control *Erg*$^{fl/fl}$ and *Erg*$^{cEC\text{-}KO}$ embryos at gestation ages E9.5, E10.5 and E11.5. Scale bar, 500 µm. (**F**) Confocal microscopy of E10.5 *Erg*$^{fl/fl}$ and *Erg*$^{cEC\text{-}KO}$ embryos labelled with antibodies to endomucin (green) and ERG (red) in blood vessels surrounding the developing eye. Scale bar, 50 µm. (**G**) Capillary vessel detail from head regions of *Erg*$^{fl/fl}$ and *Erg*$^{cEC\text{-}KO}$ E10.5 embryos shows the presence of multiple blunt-ended capillaries (arrowheads). Scale bar, 50 µm. (**H**) PCR amplification was carried out on genomic DNA isolated from tamoxifen-treated *Erg*$^{iEC\text{-}KO}$ and *Erg*$^{fl/fl}$ mice to detect the presence of the *loxP* sequences around *Erg* exon 6 (Flox PCR), the *Cre* transgene (Cre PCR), or the intronic sequences that span *Erg* exon 6 (Cre excision PCR). Lane 1, *Erg*$^{iEC\text{-}het}$; lane 2, *Erg*$^{iEC\text{-}KO}$; lane 3, *Erg*$^{fl/+}$; lane 4, *Erg*$^{fl/fl}$. (**I**) Representative western blot analysis (top) and relative ERG expression (bottom) from heart protein lysates. (**J**) Whole mount retinas isolated at postnatal (P) day 6 from tamoxifen-treated mice were labelled for ERG (green) and isolectin B4 (IB4, red). Arteries (A) and veins (V) are indicated. (**K**) Whole mount retinas from P6 control mice (*Erg*$^{fl/fl}$) were stained with antibodies to ERG (green) and the vasculature was labelled with isolectin B4 (red); arrows indicate tip cells containing ERG-positive nuclei.

Figure S2

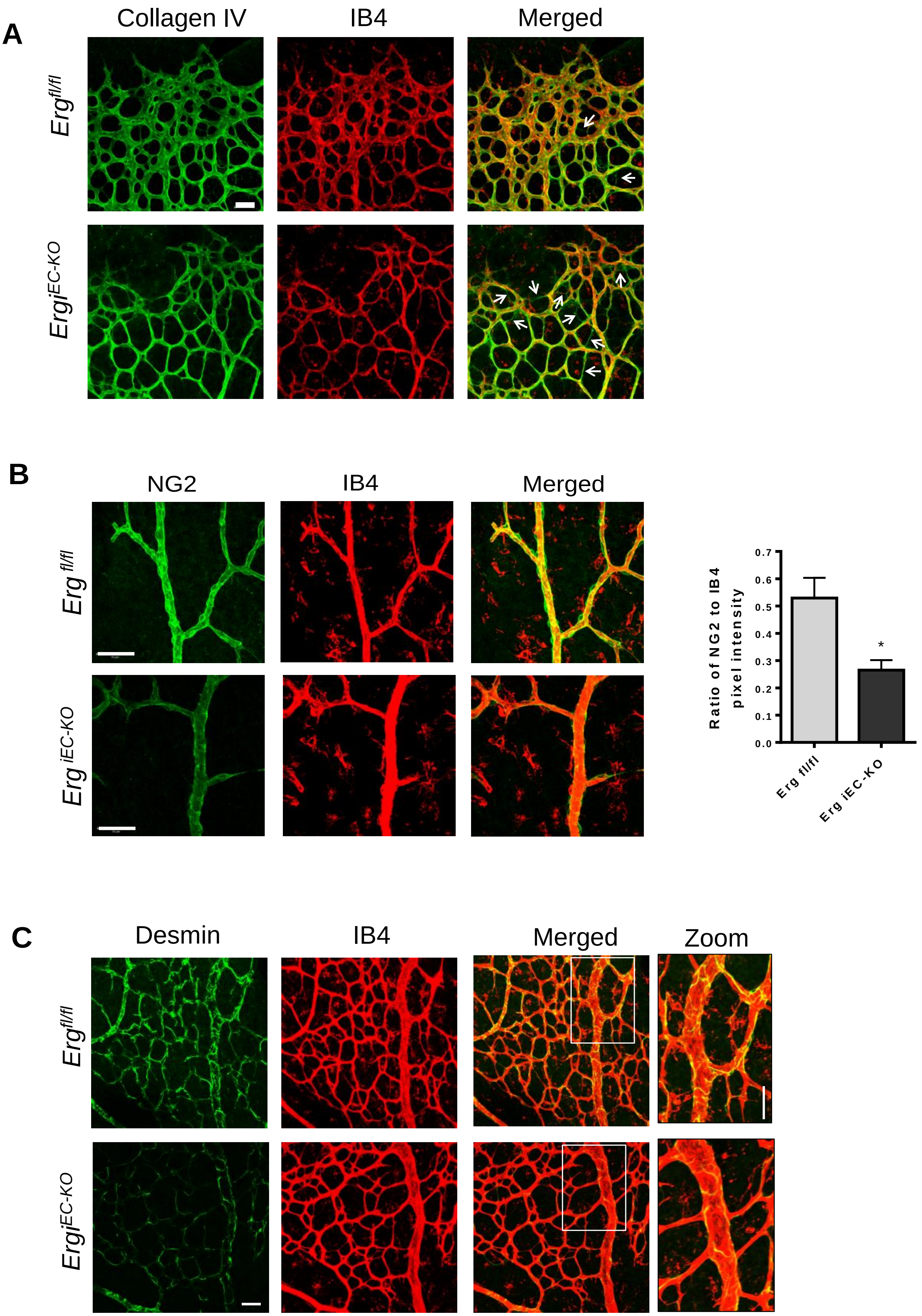
A
Collagen IV
IB4
Merged
Erg fl/fl
Erg iEC-KO
B
NG2
IB4
Merged
Erg fl/fl
Erg iEC-KO
Ratio of NG2 to IB4 pixel intensity
0.0
0.1
0.2
0.3
0.4
0.5
0.6
0.7
*
Erg fl/fl
Erg iEC-KO
C
Desmin
IB4
Merged
Zoom
Erg fl/fl
Erg iEC-KO

Figure S2, Related to Figure 2

Collagen IV, NG2 and desmin staining in the retinal vasculature of *Erg*$^{fl/fl}$ and *Erg*$^{iEC\text{-}KO}$ mice

(**A**) Staining for collagen IV (green) and isolectin B4 (IB4, red) at the angiogenic front of P6 retinas from *Erg*$^{iEC\text{-}KO}$ and *Erg*$^{fl/fl}$ mice. Arrows show empty collagen IV sleeves. (**B**) NG2-positive pericytes (green) associated with isolectin B4 labeled arteries (red) in P6 retinas, n=4. Graphical data are ± SEM, *P < 0.05. (**C**) Desmin-positive pericytes (green) and isolectin B4 (red) staining of retinal vasculature from P6 *Erg*$^{iEC\text{-}KO}$ and *Erg*$^{fl/fl}$ mice. Scale bars, 50 µm.

Figure S3

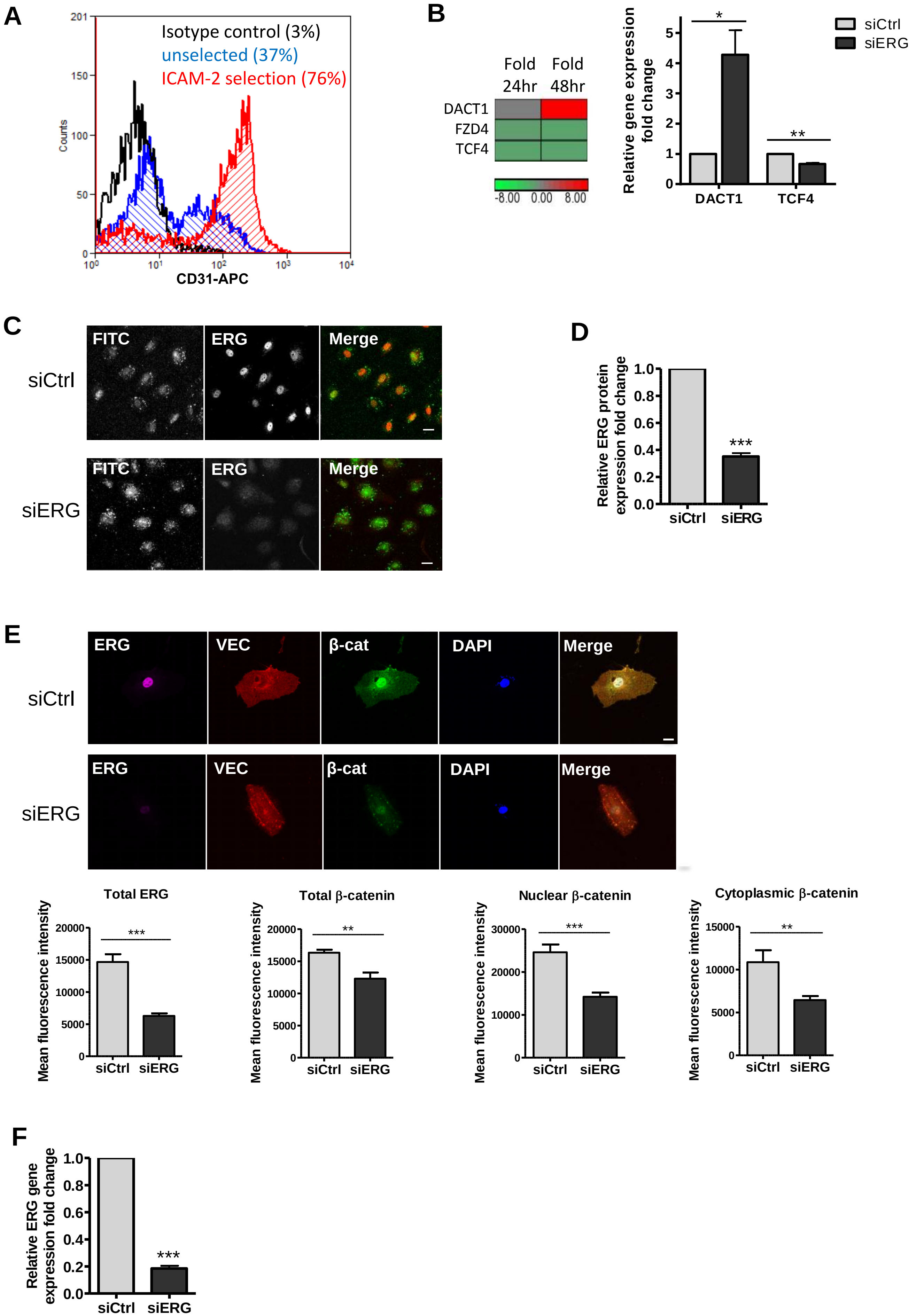

A
Isotype control (3%)
unselected (37%)
ICAM-2 selection (76%)
Counts
CD31-APC
B
Fold 24hr
Fold 48hr
DACT1
FZD4
TCF4
-8.00 0.00 8.00
Relative gene expression fold change
siCtrl
siERG
DACT1
TCF4
C
FITC
ERG
Merge
siCtrl
siERG
D
Relative ERG protein expression fold change
E
ERG
VEC
β-cat
DAPI
Merge
Total ERG
Total β-catenin
Nuclear β-catenin
Cytoplasmic β-catenin
Mean fluorescence intensity
F
Relative ERG gene expression fold change

Figure S3, Related to Figure 3

Characterisation of primary mouse lung endothelial cells; validation of siCtrl and siERG treatment in HUVEC

(**A**) Flow cytometric analysis for the endothelial marker CD31 in freshly isolated lung cells (unselected) and ICAM-2 positively selected lung cells isolated from *Erg*$^{fl/fl}$ mice (n=4). (**B**) Microarray analysis of differential gene expression in HUVEC was performed at 24 and 48 hours after Erg inhibition (Birdsey et al., 2012), with fold change of selected genes represented as high (red) and low (green) expression compared to the median (grey). qPCR analysis of mRNA levels of DACT1 and TCF-4 in 48 hour siCtrl and siERG-treated HUVEC, normalized to GAPDH and expressed relative to siCtrl (n = 4). (**C**) Immunofluorescence microscopy of FITC-conjugated siCtrl (green) and siERG-treated HUVEC. Cells were co-stained for ERG (red), scale bar, 20 µm. (**D**) Quantification of western blot analysis of ERG expression in siCtrl and siERG HUVEC (n=4). (**E**) ERG (magenta), VE-cadherin (VEC; red), β-catenin (β-cat; green) and DAPI (blue) staining of sparse control and ERG-deficient HUVEC. Scale bar, 20 µm. Volocity® software quantification of mean ERG and β-catenin fluorescence intensity. Quantification of mean β-catenin fluorescence intensity in the cytoplasm required the exclusion of nuclear areas (objects that touch DAPI), whereas quantification of mean β-catenin intensity in the nucleus required the exclusion of cytoplasmic areas (objects that do not touch or overlap with DAPI) (n=8). **(F**) ERG mRNA levels from siCtrl and siERG-treated HUVEC were quantified using qPCR, normalized to GAPDH (n=3). All graphical data are ± SEM, *P < 0.05, **P < 0.01, ***P < 0.001.

Figure S4

A

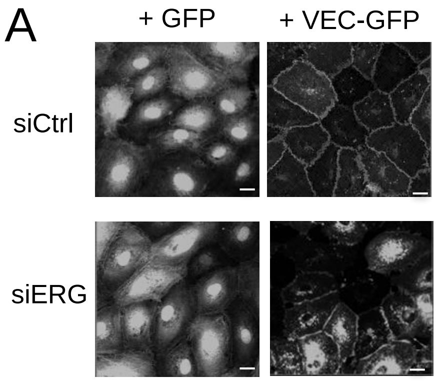

B

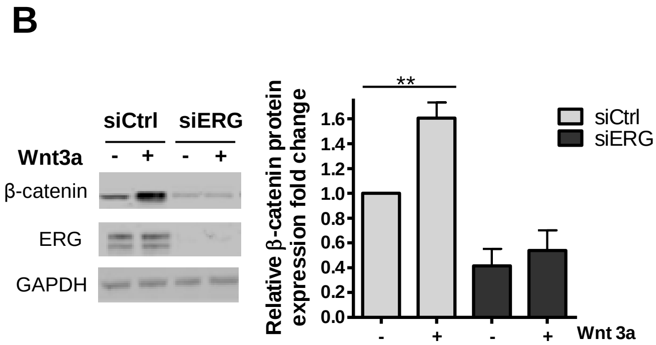

C

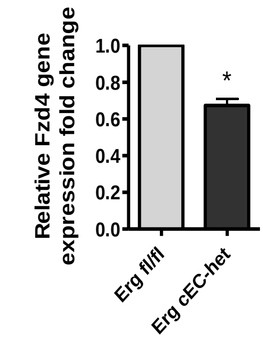

D

```
                                                                ERG-A
human   TCCCCTCCCGCCCTCTCACCCTCCCAACCTTATTTAGAAACCGTGTCCCCGAGACAGGAA -812
mouse   TTCCCTCCCGCGCTCTTACCCTCCCAACCTTATTTAGAAACCGTGTCCCCGAGACAGGAA
        * ********* **** *******************************************

                                               ERG-B
human   GGGCGGCGGGCCGAGAAGCACGGAGAGTCCCGGTCTCATTTCCTTTCGAATCCCCCTGTG -752
mouse   GGGCGGCGGGCCGAGAAGCACGGAGCGTCCCGGCCTCATTTCCTTTCGAATCCCCCTGTG
        *************************.******* **************************

                          ERG-C
human   GAATTTCATTTCATACGGTGAGGAAATCAGAGCGCGAGACCGTCGGCGGGTCTGCAGCTC -692
mouse   GAATTTCATTTCGTACGGTGAGGAAATCGGAGCGCGAGCCCATCG--------GCTGCTC
        ************.***************.*********.**.***        **:****

human   CGCAGCTCTGGGGCGCCTCCCCGCTGGCGGCTGGAGGTGGGCGGCTCCCTGCGCAGGTCG -632
mouse   TGTTGCTCCGGAGCGCCTCCCCCACGGCGACTGCAGACCGGCTGCTCCCGGCGCAGGTCG
         * :**** **.********** . ****.*** **.  *** ****** **********

human   GGCGAGTCTGGGGGCGGGG--AGAGGCCGGGCCCCGATCGCCGCTGGTCCGGAGTGAAAA -572
mouse   CGCCGAGTGCGGGGCGGGGCCGGGGGGCGGGCGCCAG-CCCCACTGGCTAGAGGTGAAAT
         ** ..    *********  .*.** ***** **.. * **.****  .*..******:

human   TAGCTGTGGACTCACGCAGTACCAGGCACCATG-CTAAGCCTTCTACACTTTATCTCTAA -514
mouse   TTTCTG------CGAACTGTACCAGGCACCAGCTCTAAGCCTTTCAGGCTTTATCGCTAA
        *: ***      *...*:*************   *********  * .******* ****

human   TCCTCCCAAGTGACTTTACAGAGGAG---ACTGACGCCGAGGGGTTGAGCAATTTGCTCA -455
mouse   TCCTCCCAAGTCACTTTACGGATGAGGAACCTGACGCGGAGAGGTTGGGCAATTTGCTCA
        *********** *******.** ***    .******* ***.*****.***********

human   AGGTCACACAACCGGGACTGAAAGCCACAATCGGTGTAAAT-CCAAATCCCCAATTCTTT -398
mouse   AGGTCACACAGCCAGGATTTAAAGCCACAATCTGTGTAAACCCCAAATACCGTGTTCTTT
        **********.**.*** * ************ *******  ******.** :.******

human   GTGTACGTTTCTCTGCCTGACGGGGAGGTGGCCACCGG-CCT-AGGTTACAGCTGAAAGG -339
mouse   GTGTTCATTATTTTGCTTGATACAGAGGTGGCCACGGGGCCGGAGGTGACAGCTGTAAGG
        ****:*.**: * *** *** . .*********** ** **  **** *******:****

            ERG--D  ERG-E
human   AGACAGGAAGGAAAGGGGGCAGATGGAACAGGGCGCACTTCTCACCTG-CAGAAGGGGGC -281
mouse   GGAAGGACGTGAAAGGGG---CATAGATGTGGGAGCACGTCTCACCTGAAAGAGAAGAGC
        .**..*... ********    **.**: :***.**** ********* .***...*.**

human   AGGACGGCGTGGCTGGAGCCACTGGGCTGCGTCCCTGCTTGGCCCCTGCGCGCCTAG-AT -222
mouse   AGGTTGGCTGGGCAGGAGCCACGGGGCTCCATCGCCGCCTGGCCACAGCGTGCAATGGAT
        ***: ***  ***:******** ***** *.** * ** *****.*:*** **.::* **

                ERG-F
human   CCGGGTGGGAGGAAAGCAAGAGCCGGGAGG-CGGGCGGGGCGGGGCGCTAGGGGAGGGAG -163
mouse   CCGGGTGGGAGGACGCCGAGCGCCGGGAAGCCAAGCGGGGCGGGGCGCCTCTGGAGCGAG
        *************.. *.**.*******.* *..************** :  **** ***

human   GGAGGGAGGGGCGGCCGGAGGAGGGGGCCGGAGGGCGGGGGCCTGACTCCTCCTGCGGGA -122
mouse   CAAGGGAGAG--------AGGATCAAGCCG-CGGGCGGGGGCCTGACCCTTACAGCAGGA
         .******.*        ****  ..**** .************** * *.*:**.***
```

Figure S4, Related to Figure 4

ERG regulation of β-catenin signaling in endothelial cells through VE-cadherin and Frizzled-4

(**A**) GFP autofluorescence was visualized using confocal microscopy of control and ERG-deficient HUVEC transduced with GFP-tagged control or VE-cadherin adenovirus (VEC-GFP). Scale bar, 20 µm (n=3). (**B**) Western blot analysis of β-catenin expression in extracts of control and ERG-deficient cells treated with β-catenin stabilizer Wnt ligand 3a (Wnt3a) (n=3). (**C**) Fzd4 mRNA expression in primary $\mathit{Erg}^{cEC\text{-}het}$ mouse lung EC compared to control (n=6). (**D**) Sequence comparison of genomic region upstream of the Fzd4 transcription start site in human and mouse. ERG consensus sequences, (A/C)GGAA(G/A) or AGGA(A/T)(G/A), are shown (conserved: green boxes, ERG A-C; non-conserved: empty boxes, ERG D-F). Asterisks denote conserved nucleotides across both species. Nucleotide numbers relative to the Fzd4 transcription start site. All graphical data are ± SEM, *P < 0.05, **P < 0.01.

Figure S5

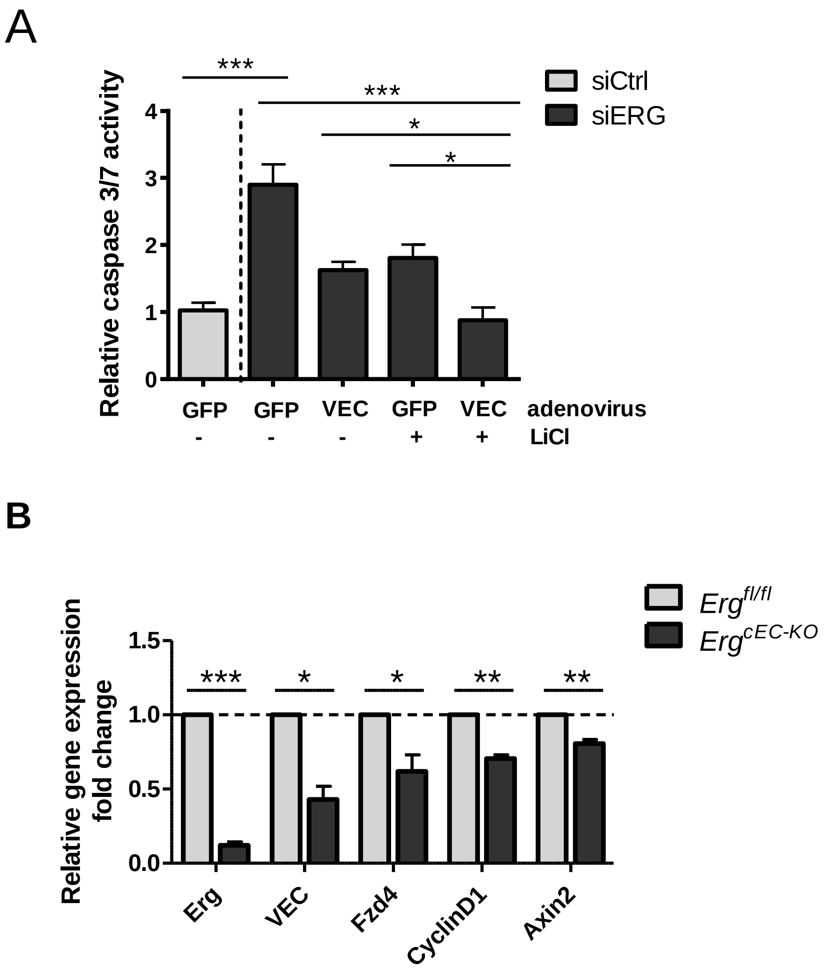

Figure S5, Related to Figure 5

ERG regulates cell survival through Wnt signaling; decreased ERG target expression in the yolk sacs from *Erg$^{cEC\text{-}KO}$* embryos

(**A**) Analysis of cell apoptosis by measuring caspase -3 or -7 activity in control or ERG-deficient cells. Cells were treated with control GFP or VE-cadherin-GFP (VEC) adenovirus in the presence and absence of LiCl (n=3). (**B**) qPCR analysis of NaCl-treated *Erg$^{fl/fl}$* and *Erg$^{cEC\text{-}KO}$* embryo yolk sacs. Data are expressed as fold change versus NaCl-treated *Erg$^{fl/fl}$* and are ± SEM from at least three mice per group. All graphical data are ± SEM, *P < 0.05, **P < 0.01, ***P < 0.001.

Figure S6

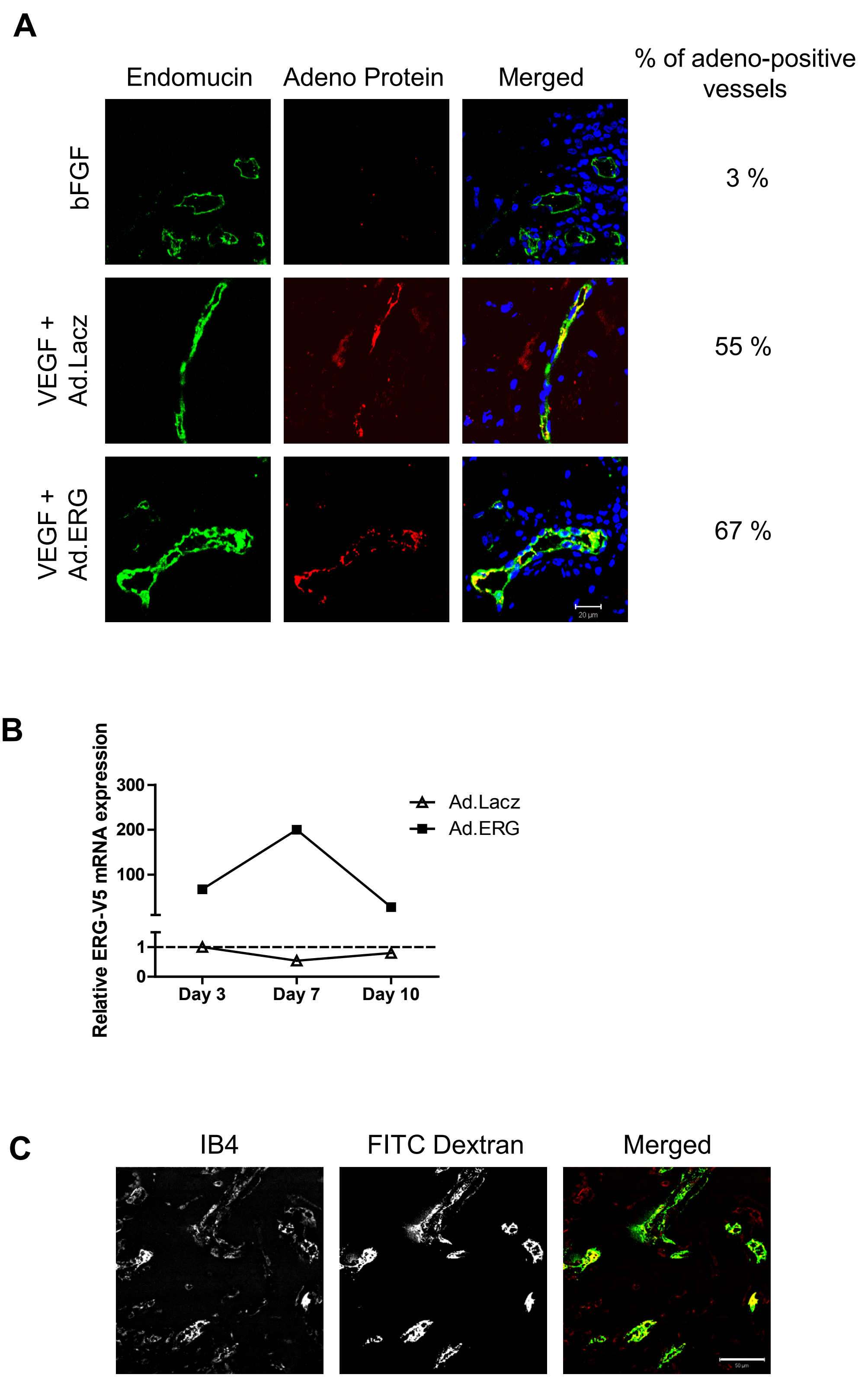

A
Endomucin
Adeno Protein
Merged
% of adeno-positive vessels
bFGF
VEGF + Ad.Lacz
VEGF + Ad.ERG
3 %
55 %
67 %
B
Relative ERG-V5 mRNA expression
300
200
100
1
0
Day 3
Day 7
Day 10
Ad.Lacz
Ad.ERG
C
IB4
FITC Dextran
Merged

Figure S6, Related to Figure 6

Validation of Ad.Lacz or Ad.ERG transduction of endothelial cells in Matrigel plugs *in vivo*
Matrigel mixture containing basic FGF, or VEGF combined with adenovirus expressing either Lacz (Ad.Lacz) or ERG (Ad.ERG), was injected subcutaneously into C57BL6 mice. (**A**) Endomucin (green), adenovirus hexon protein (red) and Draq5 (blue) staining of Matrigel cryosections. Quantification of percentage of endomucin and hexon protein-positive vessels. (**B**) Representative V5-ERG mRNA expression in sections of Matrigel plugs harvested after 3, 7 and 10 days, levels expressed relative to an Ad.LacZ sample at day 3. (**C**) Isolectin B4 (IB4, red) and FITC dextran (green) staining of cryosections from Matrigel plugs containing basic FGF.

Supplemental Videos, Related to Figure 6
3-dimensional reconstruction of neovessels inside Matrigel plugs supplemented with VEGF and adenovirus expressing either Lacz (**Video S1A**) or ERG (**Video S1B**). Perfused vessels are labeled with FITC-dextran (green) and vessel leakage is visualized with TRITC-dextran (red).

Supplemental Experimental Procedures

Mice and breeding

The targeting vector to insert *loxP* sequences around *Erg* exon 6 was constructed from a proprietary 129Sv/Pas BAC library (genOway) and PCR amplification from 129Sv/Pas ES cell genomic DNA. The linearized targeting construct was transfected by electroporation into 129Sv ES cells. Following positive selection, 334 G418-resistant ES cell clones were screened for homologous recombination of the targeting vector using both PCR and Southern blotting. Two clones were identified as being correctly targeted and these were injected into recipient blastocyts from C57BL/6J female mice. High chimeric male mice were generated and bred with C57BL/6J Flp recombinase-expressing females to remove the neomycin selection cassette and to generate heterozygous mice carrying the *Erg* floxed allele ($Erg^{fl/+}$).

Immunofluorescence analysis of mouse tissue

Embryos and yolk sacs were fixed in 4 % PFA overnight at 4 °C and then transferred to 1 % BSA, 0.5 % Triton X-100 overnight at 4 °C. Sections were incubated overnight at 4 °C with primary antibodies to rat anti-endomucin (1:100, clone V7C7, Santa Cruz) and in some cases with rabbit anti-ERG (1:200, sc-353, Santa Cruz) diluted in blocking buffer (1% BSA, 0.5 % Tween-20). Washes were carried out with PBST three times for 20 min each and then sections were incubated overnight at 4 °C with secondary antibodies to anti-rat IgG Alexa Fluor (AF) 488 and anti-rabbit IgG AF 546 (Invitrogen) diluted in blocking buffer. Following washes in PBST, embryos and yolk sacs were mounted in Fluoromount G (Southern Biotech). Confocal microscopy was carried out on a Carl Zeiss LSM510 META.

Retinas were incubated in primary antibodies to rabbit anti-ERG (1:200, Santa Cruz), and/or rabbit anti-NG2 (1:200, Millipore), mouse anti-desmin (1:100, DAKO), goat anti-Collagen IV (1:20, Millipore), rat anti-VE-cadherin (BV13, 1:50, eBioscience), biotinylated isolectin B4 (1:250, Vector Laboratories, Peterborough, UK). Secondary antibodies were anti-rabbit IgG AF 546, and/or anti-rat IgG AF 488, anti-rabbit IgG AF 488, anti-goat IgG AF 546, anti-mouse IgG AF 555, streptavidin-AF 633 (all at 1:500, Invitrogen). Confocal microscopy was carried out on a Carl Zeiss LSM510 META. Images were analyzed with ImageJ (NIH) and Volocity (PerkinElmer). For quantification of retinal vascular progression, the distance of vessel growth from the optic nerve to the periphery was measured. For vessel density, the number of vessel branch points per field was counted. For vessel regression analysis, the ratio of the numbers of isolectin B4 or endomucin-positive blood vessels to collagen IV-positive sleeves were calculated.

Matrigel cryosections were processed for immunostaining using primary antibodies to biotinylated anti-FITC (eBioscience), rabbit anti-TRITC (Abcam), mouse anti-hexon (Clonentech), FITC-isolectin B4 (Vector Laboratories), rat anti-endomucin (Santa Cruz) or mouse anti-desmin (DAKO). Secondary antibodies were streptavidin AF 555, anti-rat IgG AF 488, and anti-rabbit IgG AF 488 or anti-mouse

IgG AF 555 (all Invitrogen). Images were captured using a Carl Zeiss LSM510 META or LSM 780 confocal microscope and staining intensity was quantified using Volocity® software.

Syngeneic tumor experiments

Three month old control *Erg*$^{fl/fl}$ and *Erg*$^{iEC-KO}$ mice were injected daily with tamoxifen IP (500 µg) over 5 days prior to tumor cell injection. Mouse melanoma B16F0 (1x10^{6}) cells were injected subcutaneously into the flank of mice. After allowing the tumors to grow for 14 days, animals were killed and the tumors were excised. The tumor volume was measured using a digital calliper. Tumors were bisected and either fixed in 10 % formalin or snap-frozen in OCT solution for subsequent immunohistochemical analysis. Tumor cryosections were processed for immunostaining using primary antibodies to rat anti-endomucin (clone V7C7, Santa Cruz) and rabbit anti-NG2 (Millipore) or goat anti-collagen IV (Millipore). Secondary antibodies were anti-rat IgG AF 488 and anti-rabbit IgG AF 546 or anti-goat IgG AF 546. Blood vessel density was determined by counting endomucin-positive structures across the whole tumor section and expressed as numbers of blood vessels/mm^2.

Isolation of mouse lung endothelial cells

Primary mouse lung endothelial cells were isolated from the lungs of control *Erg*$^{fl/fl}$ and *Erg*$^{cEC-het}$ mice. Lungs were minced using GentleMACS C tubes and GentleMACS Dissociator (Miltenyi Biotec, UK), digested with 0.1% collagenase type I (Invitrogen, UK), and sieved through a 70 µm-pore cell strainer (BD Falcon, USA). Endothelial cells were selected by magnetic immunosorting (sheep anti-rat Dynabeads; Invitrogen, UK) with a negative sort for FcγRII/III receptor–positive macrophages and a positive sort for ICAM-2–positive endothelial cells. Cells were cultured in EGM-2 media (Lonza), in flasks precoated with a mixture of 0.1% gelatin (Sigma), PureCol (Invitrogen) and human plasma fibronectin (Chemicon).

Cell treatments

HUVEC were seeded onto 1 % gelatin-coated plates and grown in EGM-2 medium (Lonza,). The following day, GeneBloc antisense oligonucleotides (100 nM) or siRNA (30 nM) were transfected into HUVEC using AtuFect01 lipid (1 µg/ml, Silence Therapeutics) in EGM-2 media for 24 h or 48 h. In some experiments, HUVEC were treated overnight with lithium chloride (10 mM, Sigma) or for 6 h with MG-132 (10 µM, Calbiochem). For Wnt3a stimulation, HUVEC were transfected with siRNA for 24 h and treated for 6 h with 200 ng/ml rWnt3a (R&D).

Adenoviral transduction of HUVEC

HUVEC (5 × 10^4 cells) grown on gelatin-coated 13-mm diameter glass coverslips were transduced with adenovirus (VE-cadherin [VEC]–GFP and GFP; kindly provided by F. W. Luscinskas, Harvard Medical School, Boston, MA) as described previously (Shaw et al., 2001). After 48 h, cells were transfected with ERG or control antisense. Forty-eight hours later, cells were fixed and stained for immunofluorescence or lysates were used for immunoblotting.

Immunofluorescence analysis of HUVEC

HUVEC were cultured on gelatin-coated 13-mm diameter glass coverslips and treated with either ERG or control antisense for 48 h. In some instances, FITC-conjugated GeneBlocs were used. For analysis of sparse cells, HUVEC were treated with either siCtrl or siERG for 6 h and then reseeded at 5×10^3 cells per well onto gelatin-coated 13-mm diameter glass coverslips in a 24 well plate. Immunofluorescence labelling was carried out using the following primary antibodies: rabbit anti-ERG (Santa Cruz Biotechnology), goat anti-VE-cadherin (Santa Cruz Biotechnology) and mouse anti-active-β-catenin (Upstate-Millipore). Secondary antibodies were anti-mouse AF 488, anti-mouse AF 555, anti-rabbit AF 488, anti-rabbit AF 546, anti-goat AF 546, streptavidin AF 633 (all from Invitrogen); biotinylated anti-mouse IgG (Vector Laboratories). Nuclei were visualized using either TOPRO-3 (Invitrogen), DAPI (Invitrogen) or DRAQ5 (BioStatus, Shepshed, UK). Images were captured using a Carl Zeiss LSM510 META or LSM 780 confocal microscope. Volocity® software was used to quantify mean ERG and β-catenin fluorescence intensity. Quantification of mean β-catenin fluorescence intensity in the cytoplasm required the exclusion of nuclear areas (objects that do touch DAPI), whereas quantification of mean β-catenin intensity in the nucleus required the exclusion of cytoplasmic areas (objects that do not touch or overlap with DAPI).

Plasmid transfections and reporter assays

To measure transcriptional activity of β-catenin/TCF, cells were transfected with siRNA and after 24 h transfected with TOPFLASH or FOPFLASH reporter plasmids (Korinek et al., 1997), along with *Renilla* luciferase plasmid, using GeneJuice transfection reagent (Merck Chemicals), according to the manufacturer's conditions. TOPFLASH experiments were carried out on cells treated overnight with control, Wnt5a or Wnt3a conditioned media (CM) derived from L cells and diluted 1:1 with endothelial cell growth medium (Liebner et al., 2008). In some experiments, cells were co-transfected with a pCMV6-FZD4 expression construct (OriGene Technologies, Rockville, MD). For Frizzled-4 transactivation, a Fzd4 promoter sequence (pLightSwitchProm-Fzd4; SwitchGear, Active Motif) was subcloned into a pGL4 luciferase reporter (Promega) and co-transfected into HUVEC with *Renilla* luciferase and either an ERG-2 cDNA expression plasmid (pcDNA-ERG) or an empty plasmid (pcDNA). Luciferase reporter activity was normalized to the internal *Renilla* luciferase control and is expressed relative to control treatment using the Dual-Luciferase Reporter Assay System (Promega) and a Synergy HT microplate reader.

Fibrin gel bead assay

HUVEC treated with control or ERG siRNA in the presence or absence of LiCl (10 mM), were mixed with Cytodex 3 microcarrier beads (GE Healthcare) at a concentration of 4×10^2 cells per bead in 1.5 ml of EGM-2 medium for 4 h. The beads coated with cells were then embedded in a fibrin clot and incubated with EGM-2 complete medium in the presence of human skin fibroblast cells. Medium and treatment were renewed every other day. For quantification of *in vitro* sprouting, images of beads were captured on an IX70 Olympus microscope with a 10X objective. Images were then analyzed using ImageJ; the number of sprouts per bead was determined and sprout length was measured in arbitrary units.

BrdU in vitro proliferation assay

HUVEC were transfected with control or ERG siRNA for 8 hr in a 6-well plate and then plated in a 96-well plate at a density of 5×10^3 cells per well in M199 with 10% FBS. Cells were treated overnight with LiCl (10 mM, Sigma). Cell proliferation was determined in vitro using a BrdU proliferation ELISA kit (Roche) according to the manufacturer's instructions.

Apoptosis assay

Apoptosis was quantified by measuring caspase 3 and 7 activation, using the Caspase-Glo 3/7 Assay (Promega, Southampton, United Kingdom) on a Bio-Tek Synergy HT multidetection microplate reader.

Western blotting

Whole cell protein lysates were prepared from HUVEC using CelLytic reagent (Sigma). In some experiments, subcellular fractionation of cells into cytoplasmic and nuclear extracts was performed using the Nuclear Extract kit (Active Motif) according to the manufacturer's instructions. Immunoblotting of cell lysates was performed according to standard conditions. Immunoblots were labelled with the following primary antibodies: anti-active β-catenin (Upstate-Millipore), anti-ERG (Santa Cruz Biotechnology), anti-Fzd4 (Santa Cruz Biotechnology), anti-GAPDH (Millipore), anti-GFP (Santa Cruz Biotechnology), anti-HDAC1 (Abcam), anti-tubulin (Sigma-Aldrich) and anti-VE-cadherin (BD Biosciences). Primary antibodies were detected using fluorescently labelled secondary antibodies: goat anti-rabbit IgG DyLight 680 and goat anti-mouse IgG Dylight 800 (Thermo Scientific). Detection and quantification of fluorescence intensity were performed using an Odyssey® CLx imaging system (LI-COR Biosciences, Lincoln) and Odyssey® 2.1 software. In some instances, HRP-conjugated secondary antibodies were used for chemiluminescence detection and protein levels were quantified by densitometry and normalized against loading controls.

Bioinformatic analyses

ERG transcription factor motif discovery was performed using the JASPAR database (http://jaspar.genereg.net) (Vlieghe et al., 2006). Genome-wide ChIP-seq data for H3K4me1 and H3K27ac histone modifications and RNA polymerase II occupancy in HUVEC and sequence conservation based on Multiz alignment analysis of 100 vertebrate species were obtained from the 'ENCODE histone modification tracks' of the UCSC Genome Browser (http://genome.ucsc.edu). Mouse and human sequences were compared using ClustalW (Larkin et al., 2007; Goujon et al., 2010).

Gene set enrichment analysis (GSEA) was carried out using Gene Set Enrichment Analysis Software (GSEA, version 2) (http://www.broad.mit.edu/gsea) (Subramanian et al., 2005). The query dataset were the genes identified as being down-regulated following 24 h ERG inhibition in HUVEC (Birdsey et al., 2012), which were compared against genes identified by transcriptome analysis of 24 h β-catenin inhibition in human pulmonary artery endothelial cells (Alastalo et al., 2011). The Database

for Annotation, Visualization and Integrated Discovery (DAVID) (Huang et al., 2009) was used to identify over-represented gene ontology (GO) categories. The functional clustering tool within DAVID was used to group together GO annotations that have similar gene members and assign an enrichment score (ES). We used an ES > 1.3 (which corresponds to $P < 0.05$) to identify genes that may be over-represented in particular annotation categories.

Real-time polymerase chain reaction

RNA was extracted from tissues and HUVEC using the RNeasy kit (Qiagen). First strand cDNA synthesis was carried out using Supercript III Reverse Transcriptase (Invitrogen). Quantitative real-time PCR was performed using PerfeCTa SYBR Green Fastmix (Quanta Biosciences) on a Bio-Rad CFX96 system.

ChIP-qPCR

HUVEC previously transfected with *ERG* or control siRNA were crosslinked for 10 min with formaldehyde (to a final concentration of 1%). Chromatin was sheared using a Bioruptor UCD-200 ultrasound sonicator (Diagenode), resulting in DNA fragments of 500–1000 bp in size. Chromatin was immunoprecipitated with 2 µg antibody to ERG (sc-353, Santa Cruz Biotechnology), or negative control rabbit IgG (PP64, Chemicon, Millipore). Immunoprecipitated DNA was then used as template for quantitative PCR using primers specific for the Fzd4 genomic loci.

Oligonucleotides used in this study

Primers		Oligonucleotide Sequences
Axin 2 (human)	Forward	5′- CATTTCCCGAGAACCCACCGCC -3′
	Reverse	5′- TGTGGCGGCTCTCCAACTCCA -3′
Axin 2 (mouse)	Forward	5′- GGTCCTGGCAACTCAGTAACA -3′
	Reverse	5′- CTCATGTGAGCCTCCTCTCTTTT -3′
β-catenin (human)	Forward	5′- TGCGTGAGCAGGGTGCCATTC -3′
	Reverse	5′- CATGCGGACCCCCTCCACAA -3′
β-catenin (mouse)	Forward	5′- GTCAGTGCAGGAGGCCG -3′
	Reverse	5′- CAGGTCAGCTTGAGTAGCCA -3′
Claudin 3 (mouse)	Forward	5′- GAGTGCTTTTCCTGTTGGCG -3′
	Reverse	5′- TCCCTGATGATGGTGTTGGC -3′
Cyclin D1(human)	Forward	5′- TCAAGTGTGACCCGGACTGCCT -3′
	Reverse	5′- GCCTGGCGCAGGCTTGACT -3′
Cyclin D1 (mouse)	Forward	5′- GCGTACCCTGACACCAATCT -3′
	Reverse	5′- CACAGACCTCCAGCATCCAG -3′
Cre	Forward	5′- GCCTGCATTACCGGTCGATGCAACGA -3′
	Reverse	5′- GTGGCAGATGGCGCGGCAACACCATT -3′
Cre excision	Forward	5′- CTATGGATGAGACATGAATTTCACCATGC -3′
	Reverse	5′- AAGCACTTCCTTTGGATCACACACCTATTC -3′
DACT1 (human)	Forward	5′- ACAGTCGGCCTAGCTCAGGGTT -3′
	Reverse	5′- TGCAGATTTGGGGCAACCATCTGA -3′
ERG (human)	Forward	5′- GGAGTGGGCGGTGAAAGA -3′
	Reverse	5′- AAGGATGTCGGCGTTGTAGC -3′
ERG exon 6 (mouse)	Forward	5′- CCGGATACTGTGGGGATGAG -3′
	Reverse	5′- TCTGCGCTCATTTGTGGTCA -3′
ERG flp	Forward	5′- AGATTTTGTTCTGGTTAACAAGCCGTGC -3′
	Reverse	5′- AATGAGACAGAGCCATGAGGTAGATGGG -3′
ERG-V5	Forward	5′- CTCCAGCCCTCCCCGTGACA -3′
	Reverse	5′- TTCGAACCGCGGGCCCTCTA -3′
Frizzled4 (human)	Forward	5′- GCTCCAGCCAGCTGCAGTTCT -3′
	Reverse	5′- CGCATGGGCCAATGGGGATGT -3′
Frizzled4 (mouse)	Forward	5′- TTCGGGGACGAGGAGGAG -3′
	Reverse	5′- ACCGAACAAAGGAAGAACTGC -3′
Fzd4 R1 promoter (ChIP)	Forward	5′- TTTAGAAACCGTGTCCCCGAG -3′
	Reverse	5′- GTCTCGCGCTCTGATTTCCT -3′
Fzd4 Ctrl 3′UTR (ChIP)	Forward	5′- GCCAATCTGGGGGACTTTCA -3′
	Reverse	5′- TTCAGGGCATGTGTAGCAGG -3′
GAPDH (human)	Forward	5′- CAAGGTCATCCATGACAACTTTG -3′
	Reverse	5′- GGGCCATCCACAGTCTTCTG -3′
HPRT (mouse)	Forward	5′- GTTAAGCAGTACAGCCCCAAAATG -3′
	Reverse	5′- TCAAGGGCATATCCAACAACAAAC -3′
PLVAP(mouse)	Forward	5′- CCCTCCACCCATTGATCCAG -3′
	Reverse	5′- CAGCAGGGTTGACTACAGGG -3′
TCF-1 (human)	Forward	5′- TTCTTGGCAGAAGGTGGCAT -3′
	Reverse	5′- AGGCAGCTGTCATTCTTGGA -3′
TCF-1 (mouse)	Forward	5′- GTAAGGTCCACGGTGTACGG -3′
	Reverse	5′- TACTTGGTGTAAGGCCGCAG -3′
TCF-4 (human)	Forward	5′- GGTCAAGATCTAGCAATAATGACGA -3′
	Reverse	5′- CATCCTCCGCTCCTTCTCAC -3′

Supplemental References

Alastalo,T.P., Li,M., Perez,V.J., Pham,D., Sawada,H., Wang,J.K., Koskenvuo,M., Wang,L., Freeman,B.A., Chang,H.Y., and Rabinovitch,M. (2011). Disruption of PPARgamma/beta-catenin-mediated regulation of apelin impairs BMP-induced mouse and human pulmonary arterial EC survival. J. Clin. Invest 121, 3735-3746.

Birdsey,G.M., Dryden,N.H., Shah,A.V., Hannah,R., Hall,M.D., Haskard,D.O., Parsons,M., Mason,J.C., Zvelebil,M., Gottgens,B., Ridley,A.J., and Randi,A.M. (2012). The transcription factor Erg regulates expression of histone deacetylase 6 and multiple pathways involved in endothelial cell migration and angiogenesis. Blood 119, 894-903.

Goujon,M., McWilliam,H., Li,W., Valentin,F., Squizzato,S., Paern,J., Lopez,R (2010). A new bioinformatics analysis tools framework at EMBL-EBI. Nucleic Acids Research 38: W695-9.

Huang,d.W., Sherman,B.T., and Lempicki,R.A. (2009). Systematic and integrative analysis of large gene lists using DAVID bioinformatics resources. Nat. Protoc. 4, 44-57.

Korinek,V., Barker,N., Morin,P.J., van Wichen D., de Weger R., Kinzler,K.W., Vogelstein,B., and Clevers,H. (1997). Constitutive transcriptional activation by a beta-catenin-Tcf complex in APC-/- colon carcinoma. Science 275, 1784-1787.

Larkin,M.A., Blackshields,G., Brown,N.P., Chenna,R., McGettigan,P.A., McWilliam,H., Valentin,F., Wallace,I.M., Wilm,A., Lopez,R., Thompson,J.D., Gibson,T.J. and Higgins,D.G. (2007). ClustalW and ClustalX version 2. Bioinformatics 23(21), 2947-2948.

Liebner,S., Corada,M., Bangsow,T., Babbage,J., Taddei,A., Czupalla,C.J., Reis,M., Felici,A., Wolburg,H., Fruttiger,M., Taketo,M.M., von,M.H., Plate,K.H., Gerhardt,H., and Dejana,E. (2008). Wnt/beta-catenin signaling controls development of the blood-brain barrier. J. Cell Biol. 183, 409-417.

Shaw,S.K., Bamba,P.S., Perkins,B.N., and Luscinskas,F.W. (2001). Real-time imaging of vascular endothelial-cadherin during leukocyte transmigration across endothelium. J. Immunol. 167, 2323-2330.

Subramanian,A., Tamayo,P., Mootha,V.K., Mukherjee,S., Ebert,B.L., Gillette,M.A., Paulovich,A., Pomeroy,S.L., Golub,T.R., Lander,E.S., and Mesirov,J.P. (2005). Gene set enrichment analysis: a knowledge-based approach for interpreting genome-wide expression profiles. Proc. Natl. Acad. Sci. U.S.A 102, 15545-15550.

Vlieghe,D., Sandelin,A., De Bleser,P.J., Vleminckx,K., Wasserman,W.W., van,R.F., and Lenhard,B. (2006). A new generation of JASPAR, the open-access repository for transcription factor binding site profiles. Nucleic Acids Res. 34, D95-D97.
