# Supplementary figures and images for: The Endothelial Transcription Factor ERG Promotes Vascular Stability and Growth through Wnt/β-Catenin Signaling
Source: Dev Cell. 2015 Jan 12;32(1):82–96. doi: 10.1016/j.devcel.2014.11.016 (PMC4292982; doi:10.1016/j.devcel.2014.11.016)

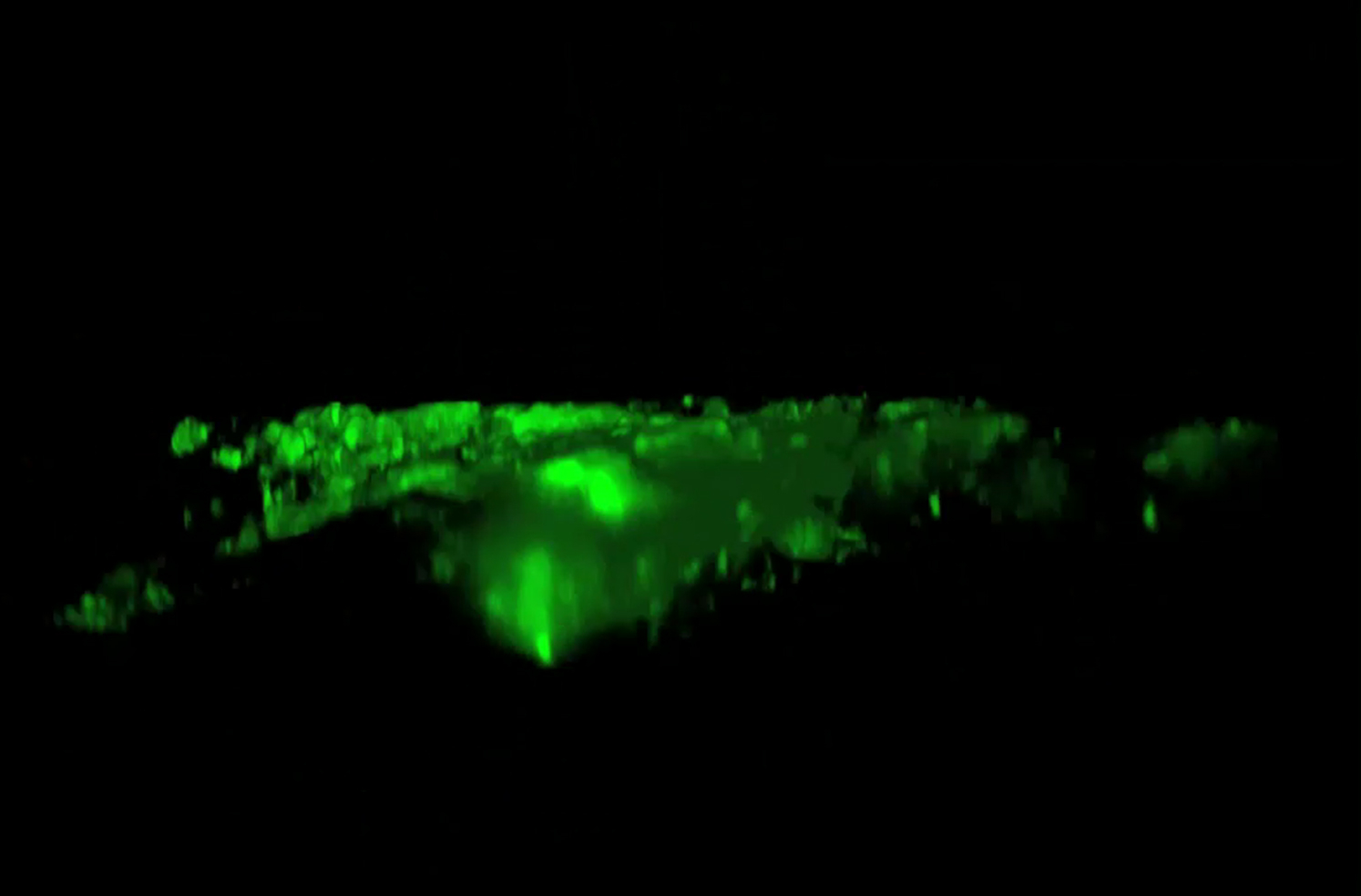

Supplement: Movie S1. 3D Reconstruction of Neovessels inside Matrigel Plugs Supplemented with VEGF and Adenovirus Expressing Lacz, Related to Figure 6 — Perfused vessels are labeled with FITC-dextran (green) and vessel leakage is visualized with TRITC-dextran (red). [file mmc2.jpg]

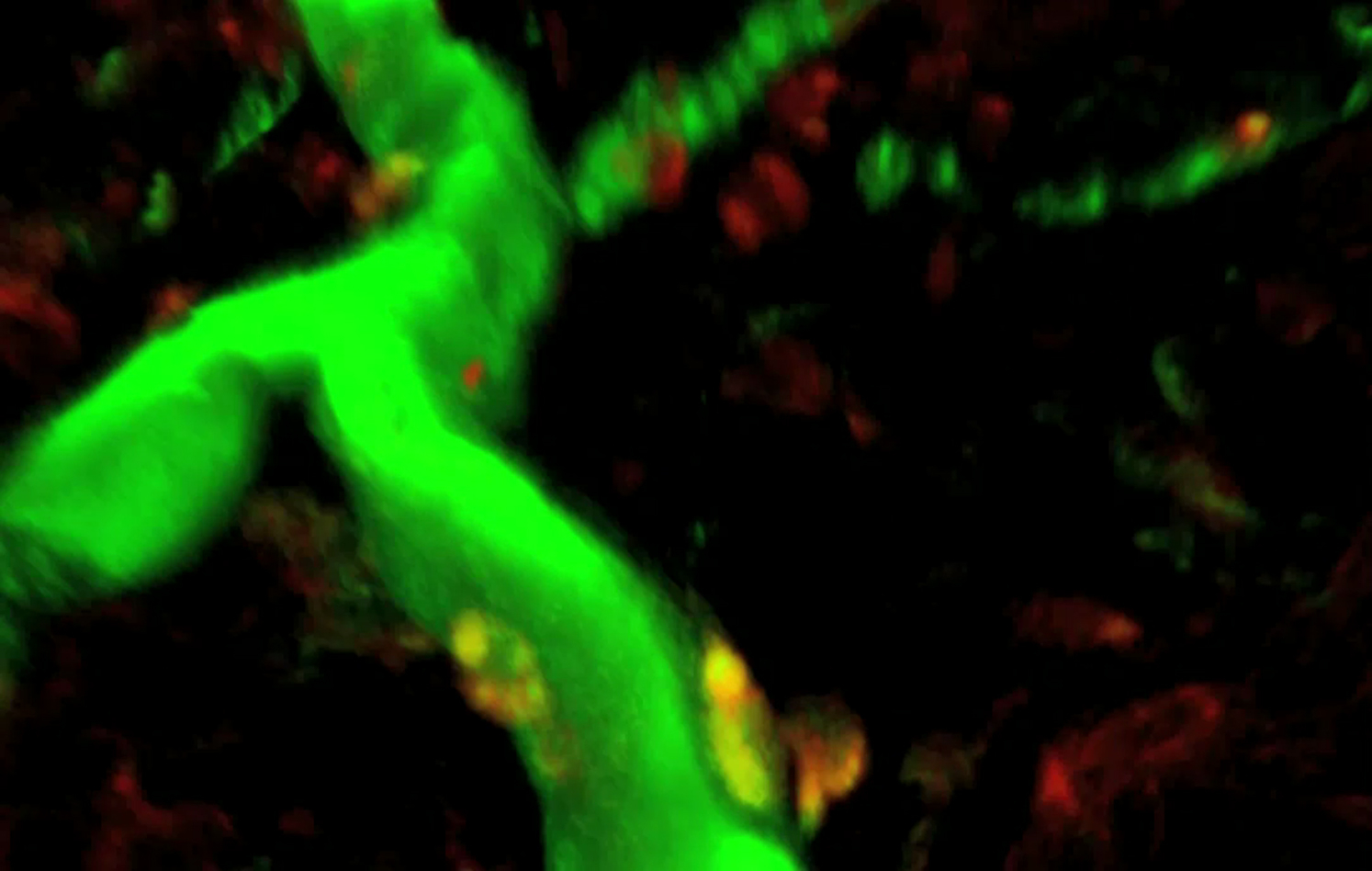

Supplement: Movie S2. 3D Reconstruction of Neovessels inside Matrigel Plugs Supplemented with VEGF and Adenovirus Expressing ERG, Related to Figure 6 — Perfused vessels are labeled with FITC-dextran (green) and vessel leakage is visualized with TRITC-dextran (red). [file mmc3.jpg]
